# Correction to: scBFA: modeling detection patterns to mitigate technical noise in large-scale single-cell genomics data

**DOI:** 10.1186/s13059-019-1822-0

**Published:** 2019-11-21

**Authors:** Ruoxin Li, Gerald Quon

**Affiliations:** 1 Graduate Group in Biostatistics, University of California, Davis, Davis, CA USA; 2 Genome Center, University of California, Davis, Davis, CA USA; 3 Department of Molecular and Cellular Biology, University of California, Davis, Davis, CA USA

**Correction to: Genome Biol**

**https://doi.org/10.1186/s13059-019-1806-0**

Following publication of the original article [1], the following two errors were found in formulae:

1) In the method section titled **“Benchmarking dimensionality reduction methods for scRNA-seq”,** the brackets should be removed from inside the square roots. The correct equation is shown below:

$$ \mathrm{MCC}=\frac{TP\ast TN- FP\ast FN}{\left(\sqrt{TP+ FP}\right)\ast \left(\sqrt{TP+ FN}\right)\ast \left(\sqrt{TN+ FP}\right)\ast \left(\sqrt{TN+ FN}\right)} $$

2) In the method section titled **“Batch effect correction”,** the position of superscript in the subscript are incorrect. The correct equation is shown below.

$$ {\mathbf{M}}_{\mathbf{sub}\left({i}^{\hbox{'}},j\right)}\sim P\left({g}^{-1}\left({\mathbf{x}}_{\mathbf{sub}\left({\mathbf{i}}^{\hbox{'}}\right)}^{\mathbf{T}}{\boldsymbol{\upbeta}}_{\mathbf{j}}+{\mathbf{z}}_{\mathbf{sub}\left({\mathbf{i}}^{\hbox{'}}\right)}^{\mathbf{T}}{\mathbf{a}}_{\mathbf{j}}+{u}_{\mathrm{sub}\left({i}^{\hbox{'}}\right)}+{\upsilon}_j\right)\right) $$
