# Supplementary material for: scBFA: modeling detection patterns to mitigate technical noise in large-scale single-cell genomics data
Source: Genome Biol. 2019 Sep 9;20:193. doi: 10.1186/s13059-019-1806-0 (PMC6734238; doi:10.1186/s13059-019-1806-0)
Supplement: Supplementary file 1 — Contains supplementary figures and tables, Figures S1–S29., Tables S1–S8. (DOCX 3090 kb) [file 13059_2019_1806_MOESM1_ESM.docx]

**Supplementary Materials for**

**scBFA: modeling detection patterns to mitigate technical noise in large-scale single cell genomics data**

Ruoxin Li^1,2^, Gerald Quon^1,2,3,*^

^1^Graduate Group in Biostatistics, ^2^Genome Center, ^3^Department of Molecular and Cellular Biology, University of California, Davis, Davis, CA

*To whom correspondence should be addressed: gquon@ucdavis.edu

Table of Contents

[Fig S1: Gene detection rate decreases as the number of cells sequenced increases, under HVG selection. 3](#_Toc17058819)

[Fig S2: 2D tSNE visualization of the Dendritic benchmark of Shalek et al. 4](#_Toc17058820)

[Fig S3: 2D tSNE visualization of the MGE benchmark of Mayer et al. 5](#_Toc17058821)

[Fig S4: scBFA performance is robust with respect to selection of regularization parameters, under HVG selection. 6](#_Toc17058822)

[Fig S5: Performance of low dimensional embeddings with respect to cell type identification, under HEG selection. 7](#_Toc17058823)

[Fig S6: scBFA trained on the observed count matrix outperforms scBFA trained on an imputed count matrix, with respect to cell type identification. 8](#_Toc17058824)

[Fig S7: Simulation framework trained on the LPS benchmark generates simulated data that resemble the benchmark data. 9](#_Toc17058825)

[Fig S8: Simulation framework trained on the HSPC benchmark generates simulated data that resemble the benchmark data. 10](#_Toc17058826)

[Fig S9: Simulation framework trained on the LSK benchmark generates simulated data that resemble the benchmark data. 11](#_Toc17058827)

[Fig S10: scBFA outperforms quantification models when the detection noise is smaller than the quantification noise (HVG, $r=5$). 12](#_Toc17058828)

[Fig S11: scBFA outperforms quantification models when the detection noise is smaller than the quantification noise (HVG, $r=0.5$). 13](#_Toc17058829)

[Fig S12: scBFA outperforms quantification models when gene detection noise is smaller than quantification noise (HEG, $r=1$). 14](#_Toc17058830)

[Fig S13: scBFA outperforms quantification models when gene detection noise is smaller than quantification noise (HEG, $r=0.5$). 15](#_Toc17058831)

[Fig S14: scBFA outperforms quantification models when gene detection noise is smaller than quantification noise (HEG, $r=5$). 16](#_Toc17058832)

[Fig S15: Comparison of the variance in embedding dimensions of the ERCC dataset 17](#_Toc17058833)

[Fig S16: Distribution of the fraction of reads of each cell mapping to mitochondrial genes in the Dendritic benchmark of Shalek et al. 18](#_Toc17058834)

[Fig S17: 2D tSNE visualization of the Dendritic benchmark of Shalek et al. 19](#_Toc17058835)

[Fig S18: 2D tSNE visualization of the Dendritic benchmark of Shalek et al. 20](#_Toc17058836)

[Fig S19: scBFA is better informed by cell type markers than quantification models, under HEG selection. 21](#_Toc17058837)

[Fig S20: scBFA improves trajectory inference of the method Slingshot compared to other dimensionality reduction methods. 22](#_Toc17058838)

[Fig S21: scBFA accurately recovers cell type identity in scATAC-seq benchmarks. 23](#_Toc17058839)

[Fig S22: 2D tSNE visualization of the scATAC-seq benchmark GSE96769. 24](#_Toc17058840)

[Fig S23: 2D tSNE visualization of the scATAC-seq benchmark GSE74310. 25](#_Toc17058841)

[Fig S24: 2D tSNE visualization of the scATAC-seq benchmark GSE107816. 26](#_Toc17058842)

[Fig S25: Binary PCA performance versus quantification models under HEG selection. 27](#_Toc17058843)

[Fig S26: Binary PCA performance versus quantification models under HVG selection. 28](#_Toc17058844)

[Fig S27: Fast scBFA approximation is one of the fastest dimensionality reduction methods. 29](#_Toc17058845)

[Fig S28: Schematic of scBFA. 30](#_Toc17058846)

[Fig S29: The main components of the generative process of the scRNA-seq simulation framework 31](#_Toc17058847)

[Table S1: Summary of scRNA-seq cell type identification benchmark datasets. 32](#_Toc17058848)

[Table S2: Summary of gene intersection between the top 2,000 genes selected by HVG and HEG 33](#_Toc17058849)

[Table S3: Cell surface marker list for the PBMC benchmark. 34](#_Toc17058850)

[Table S4: Cell surface marker list for the HSC benchmark. 35](#_Toc17058851)

[Table S5: Cell surface marker list for the Pancreatic benchmark. 36](#_Toc17058852)

[Table S6: Group I and Group II benchmark list. 37](#_Toc17058853)

[Table S7: Summary of representative scRNA-seq datasets. 38](#_Toc17058854)

[Table S8: Summary of benchmarks used in trajectory inference 40](#_Toc17058855)


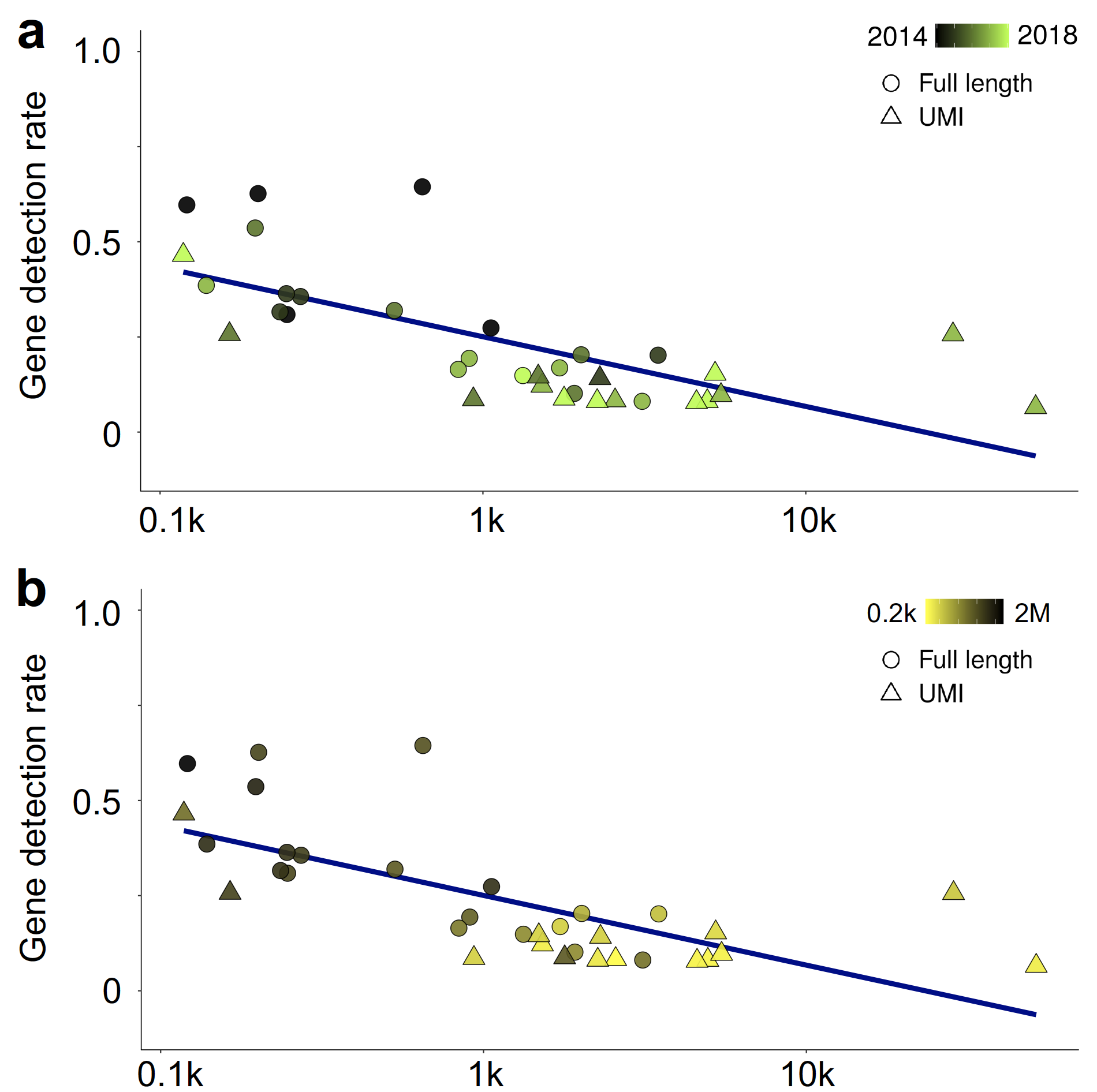


Fig S1: Gene detection rate decreases as the number of cells sequenced increases, under HVG selection. (**a**) Gene detection rate as a function of the number of cells sequenced, across 36 scRNA-seq datasets (**Table S1**). Datasets are colored by date of publication. (**b**) Same as (a), but studies are colored by library size.


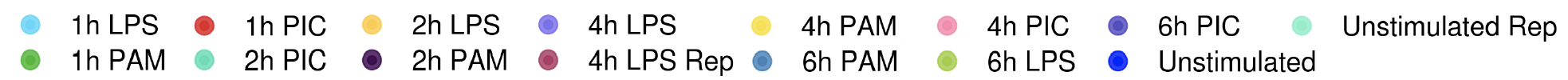

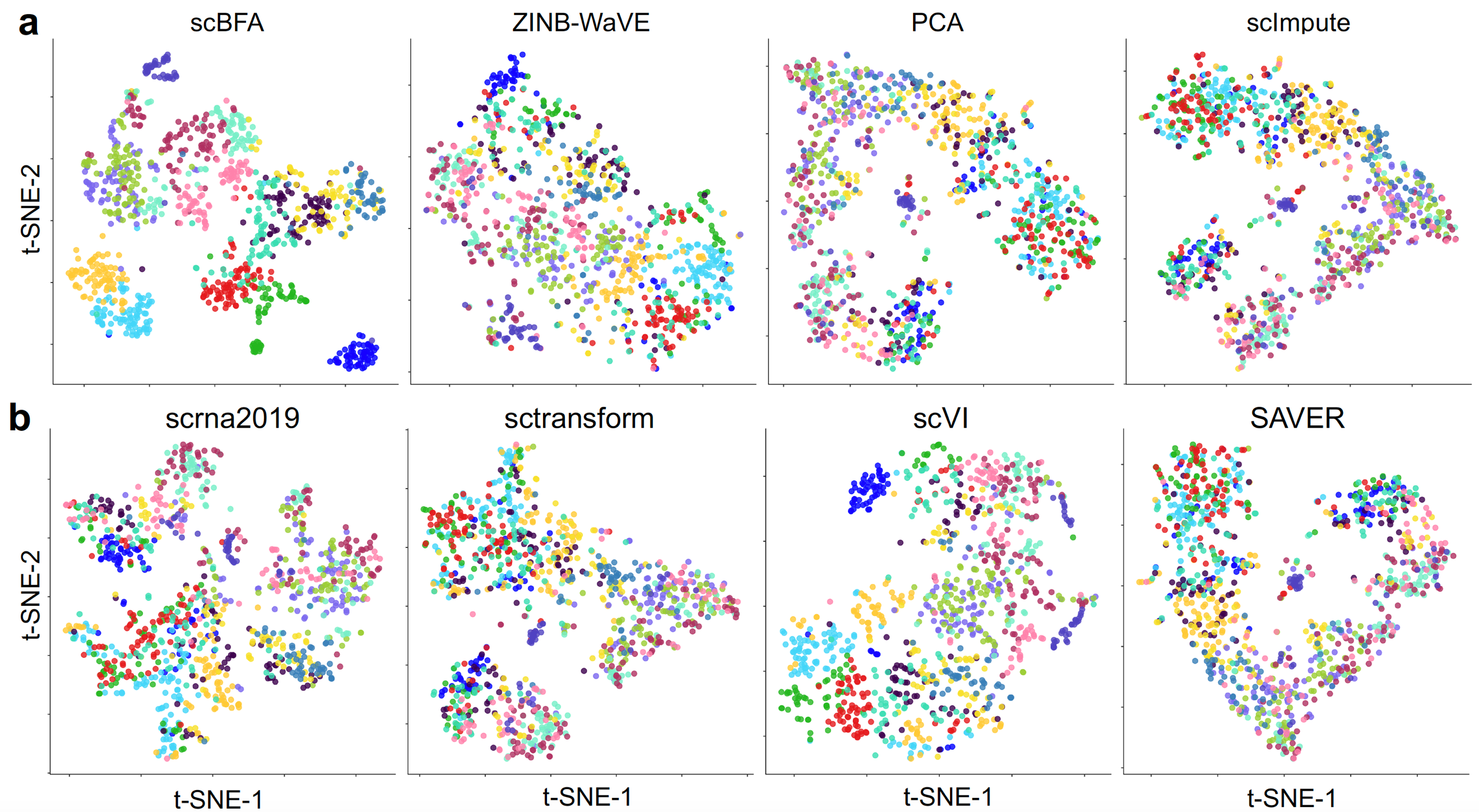


Fig S2: 2D tSNE visualization of the Dendritic benchmark of Shalek et al. tSNE plots are generated based on the 10-dimensional embeddings learned by scBFA, PCA, ZINB-WaVE, scImpute, scrna2019, sctransform, scVI and SAVER. Cells are colored by their corresponding cell types.


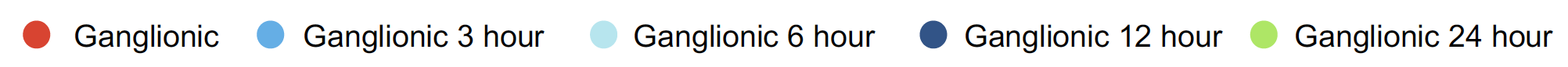


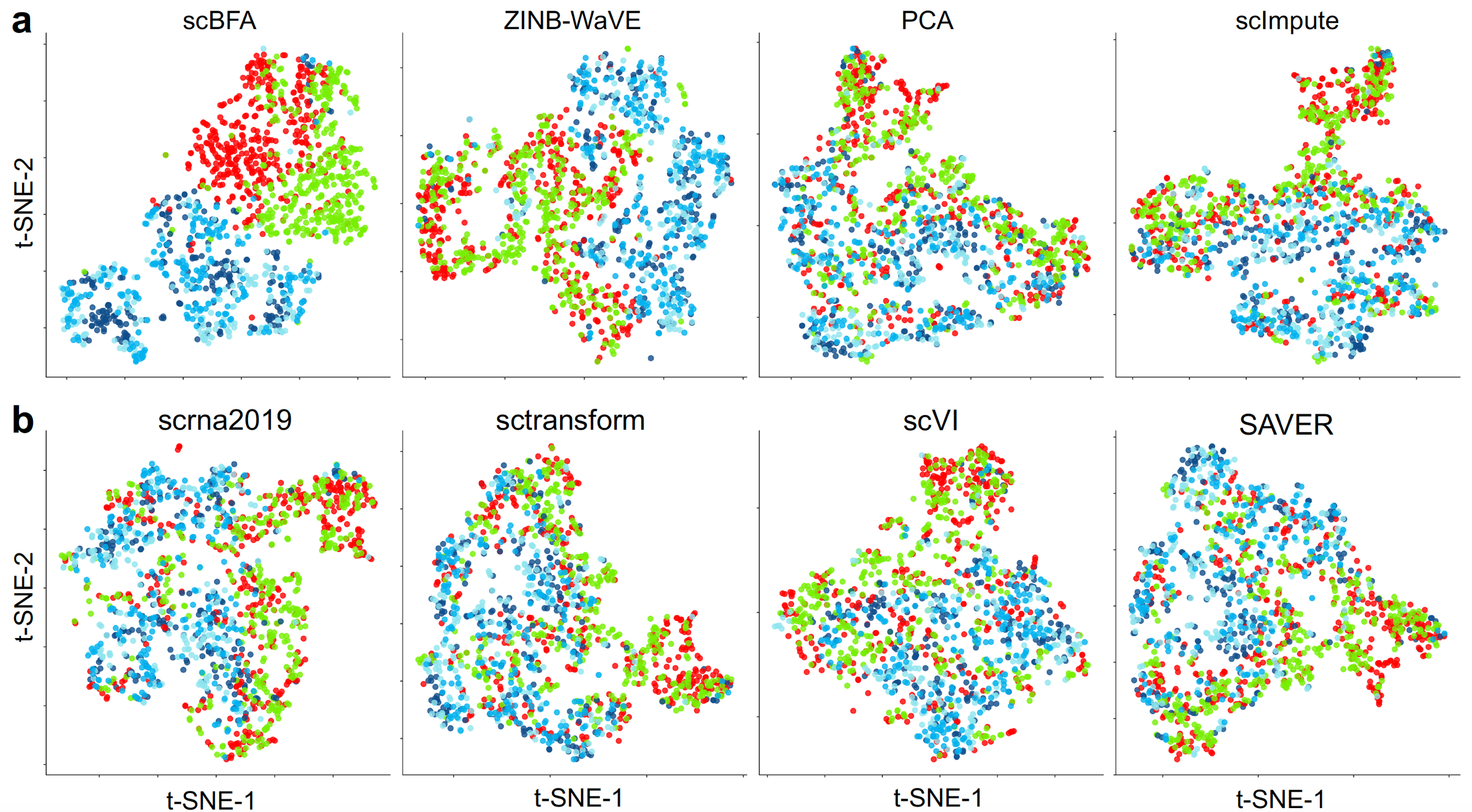


Fig S3: 2D tSNE visualization of the MGE benchmark of Mayer et al. tSNE plots are generated based on the 10-dimensional embeddings learned by scBFA, PCA, ZINB-WaVE, scImpute, scrna2019, sctransform, scVI and SAVER. Cells are colored by their corresponding cell types.


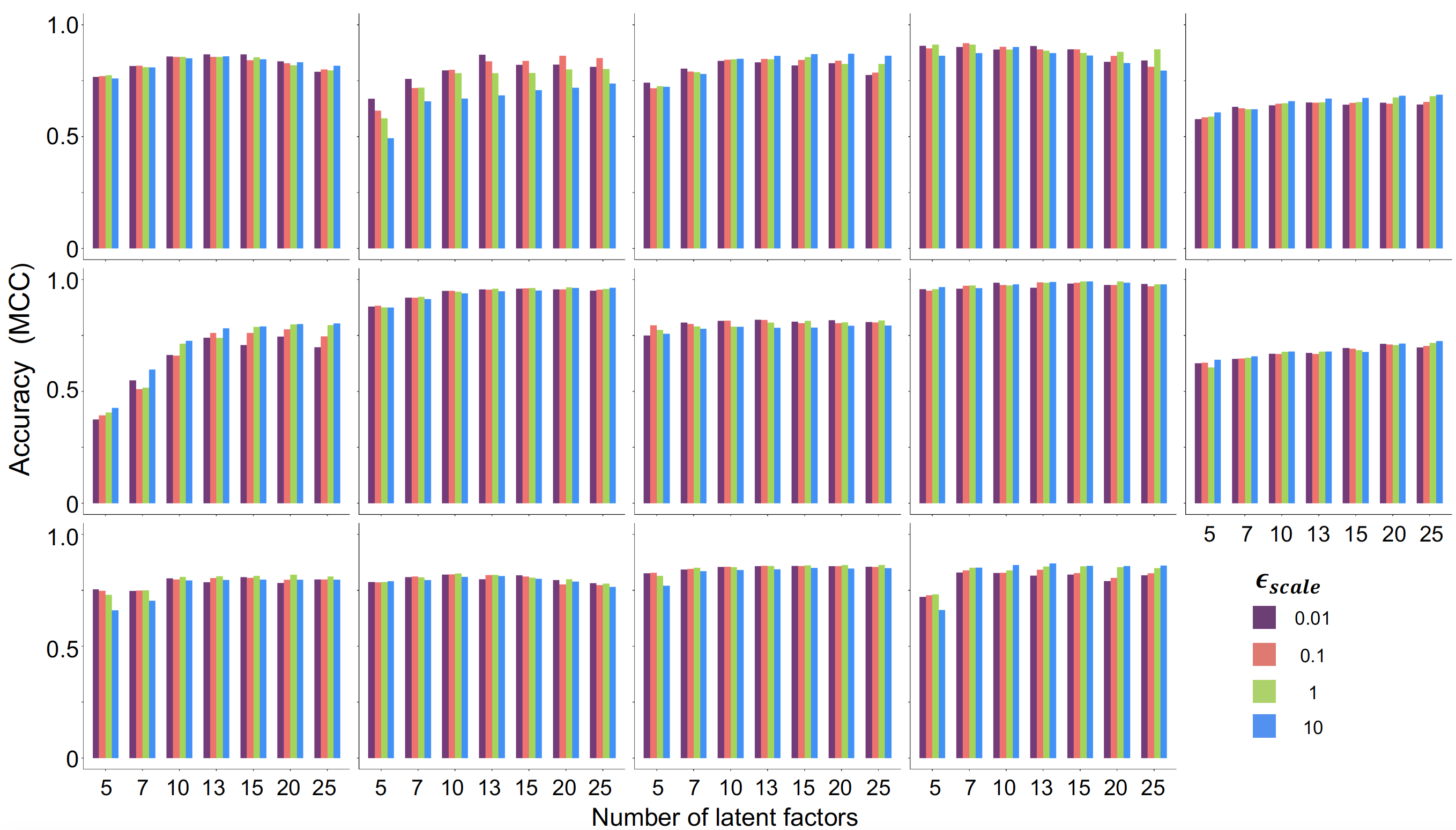


Fig S4: scBFA performance is robust with respect to selection of regularization parameters, under HVG selection. Performance is measured via cross-validation of cell type classifiers trained on scRNA-seq benchmarks in the respective embedding spaces of scBFA under different regularization settings. Each color bar represents one selection of $\epsilon_{scale}$, indicating different levels of strength of regularization given to the learned model parameters. Here the universal regularization parameter $\epsilon=\epsilon_{scale}*max(G,N)$, where we have set $\epsilon_{scale}\in\{0.01, 0.1, 1, 10\}$. Benchmarks from left to right, top to bottom: Dendritic, Pancreatic, DC, mESCs, HSPC, MGE, Intestinal, MEM-T, H7-ESC, LSK, Myeloid, HSCs, PBMC, and LPS.


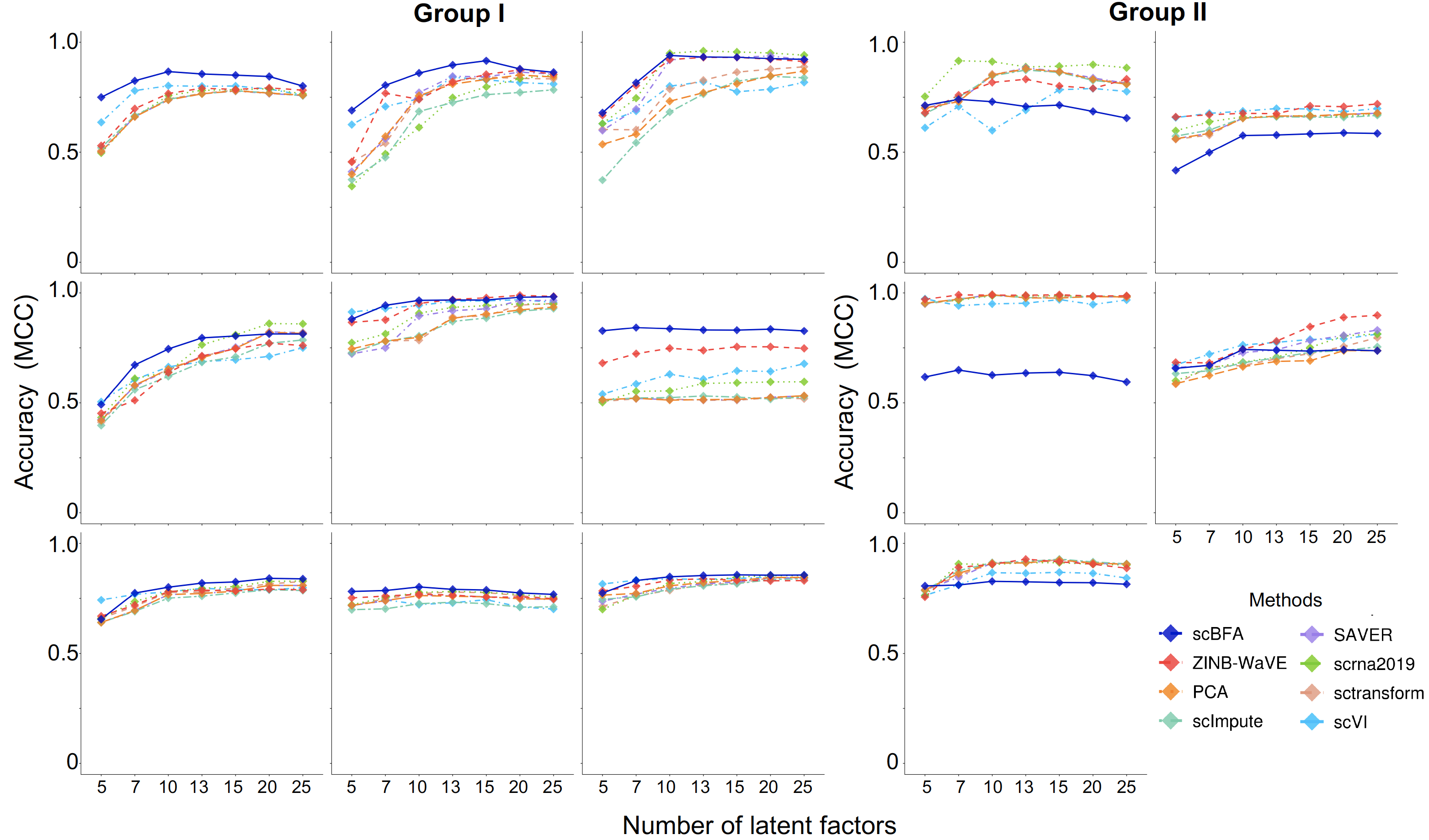


Fig S5: Performance of low dimensional embeddings with respect to cell type identification, under HEG selection. Performance is measured via cross-validation of cell type classifiers trained on scRNA-seq benchmark data in the respective embedding spaces of each method, as a function of the number of latent dimensions specified. Benchmarks are grouped based on whether scBFA is a top performer (Group I) or performs poorly (Group II). The set of Group I benchmarks from left to right, top to bottom: Dendritic, Pancreatic, DC, MGE, Intestinal, MEM-T, Myeloid, HSCs and PBMC. The set of Group II benchmarks from left to right, top to bottom: mESCs, HSPC, H7-ESC, LSK and LPS.


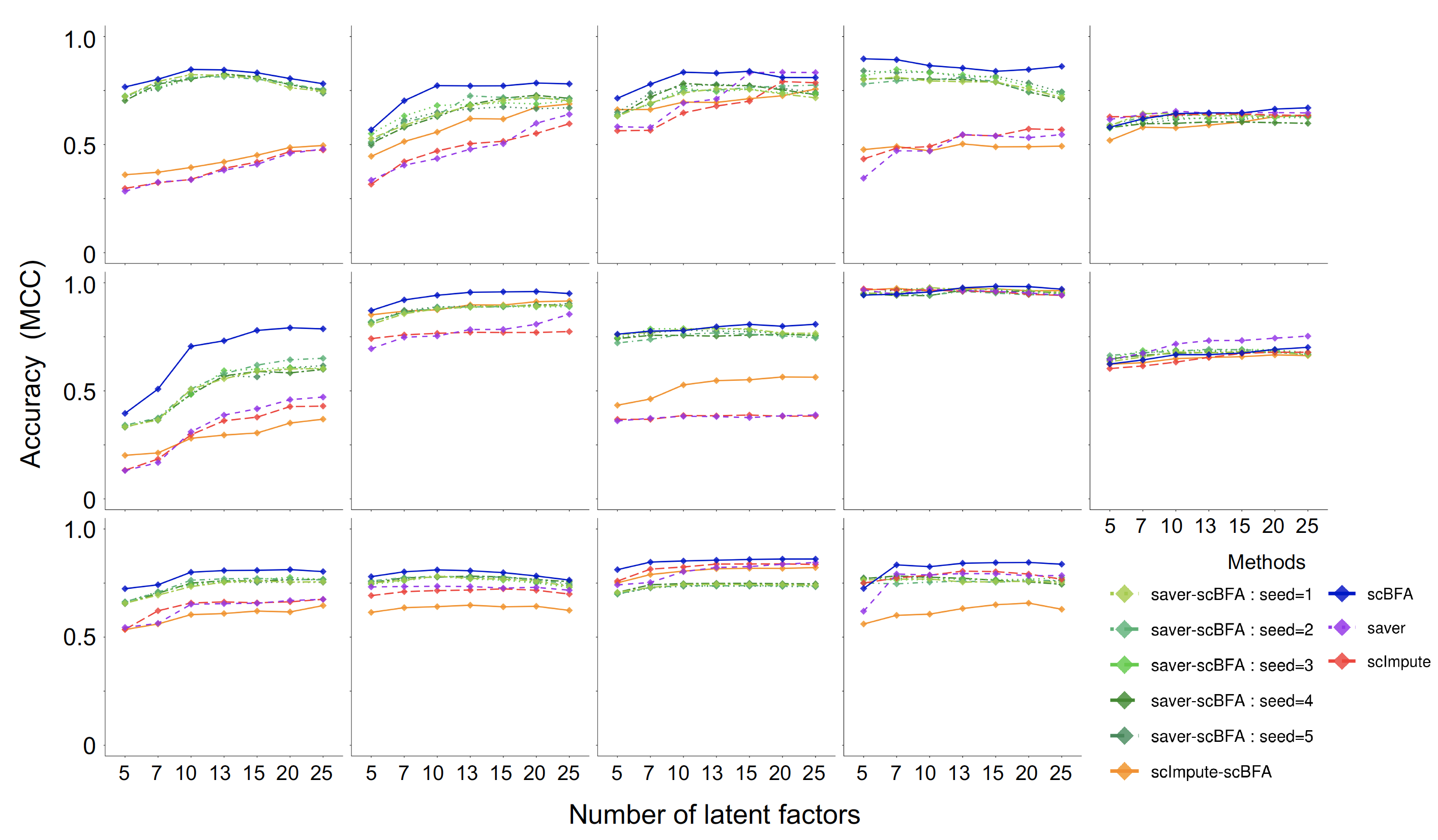


Fig S6: scBFA trained on the observed count matrix outperforms scBFA trained on an imputed count matrix, with respect to cell type identification. Performance is measured via cross-validation of cell type classifiers trained on scRNA-seq benchmark data in the respective embedding spaces of each method, as a function of the number of latent dimensions specified. Across all 14 benchmarks, scBFA performs best without imputation compared to scBFA trained on an imputed expression matrix generated from either SAVER or scImpute.


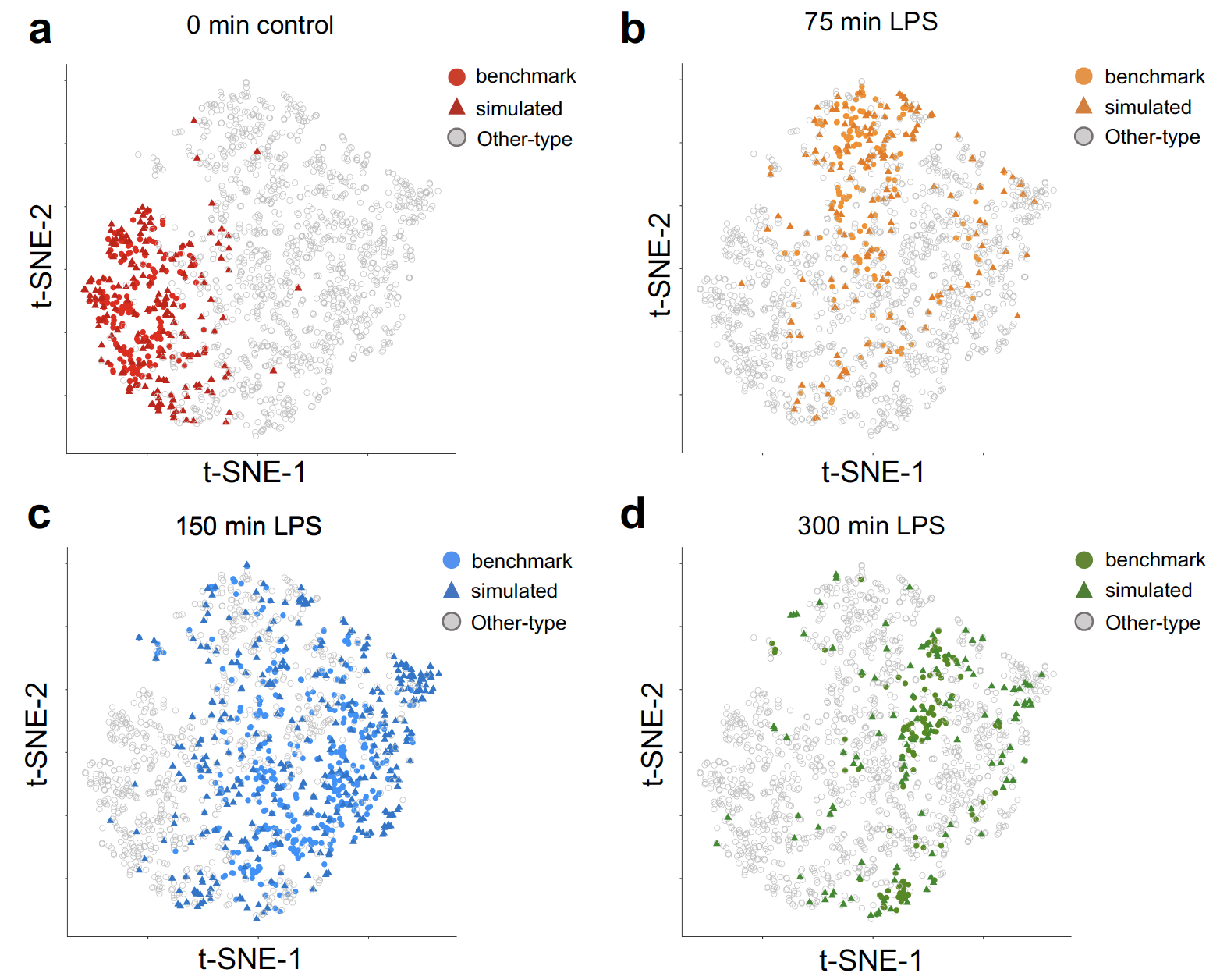


Fig S7: Simulation framework trained on the LPS benchmark generates simulated data that resemble the benchmark data. The simulation framework was used to generate a simulated dataset of the same size as the LPS benchmark with HVG selection. The combined dataset (simulated and benchmark) were jointly projected into five dimensions via PCA, then drawn in 2D using tSNE. Cells are colored according to their corresponding cell type; the cell type of each simulated cell is based on the embedding parameter learned for the corresponding cell in the benchmark. Circles indicate cells from the benchmark, while triangles represent cells from the simulated dataset. Each plot illustrates the overlap of the same cell type across the benchmark and simulated datasets, while all other cell types are shown in grey. (**a**) 0 minute control. (**b**) 75 minutes post-LPS exposure. (**c**) 150 minutes post-LPS exposure. (**d**) 300 minutes post-LPS exposure.


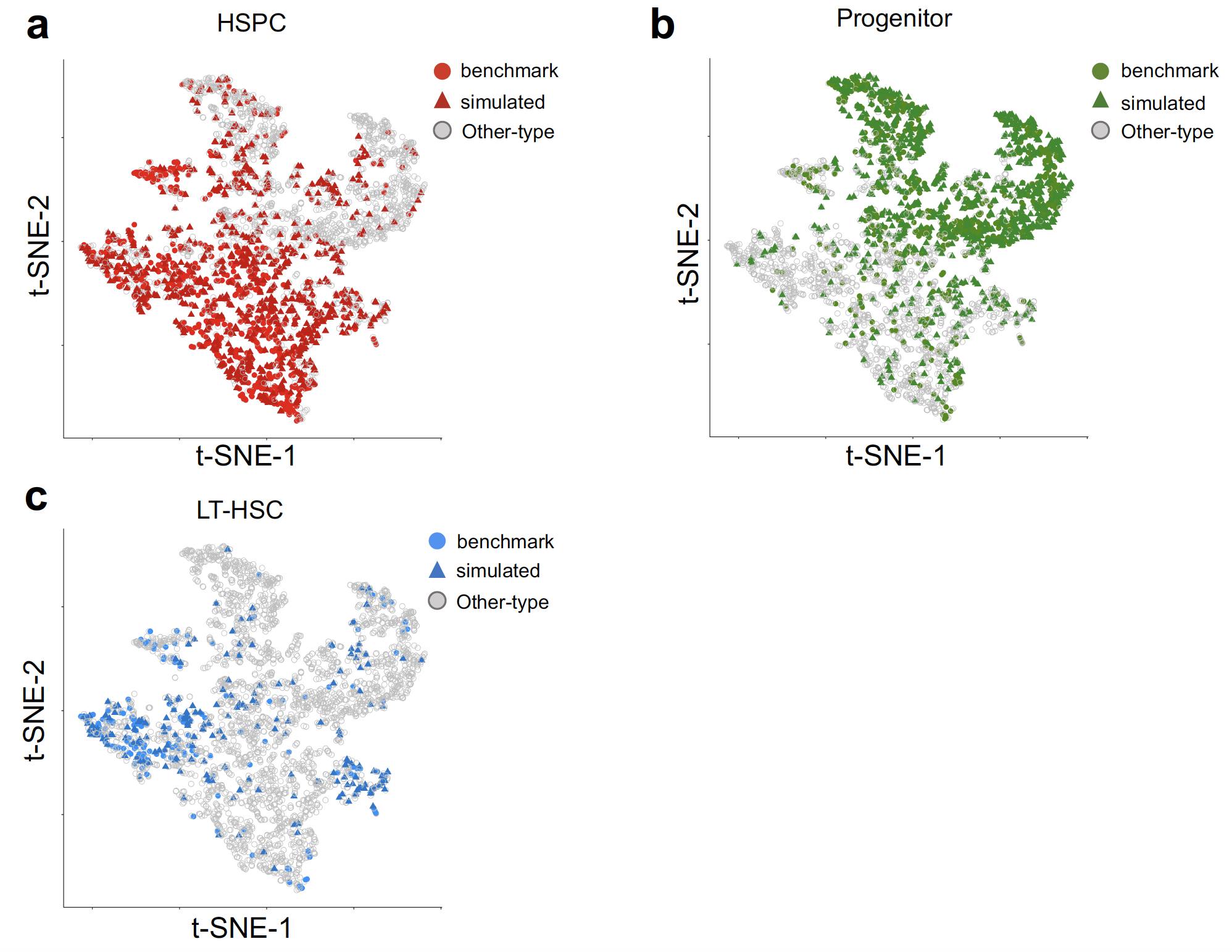


Fig S8: Simulation framework trained on the HSPC benchmark generates simulated data that resemble the benchmark data. The simulation framework was used to generate a simulated dataset of the same size as the HSPC benchmark with HVG selection. The combined dataset (simulated and benchmark) were jointly projected into five dimensions via PCA, then drawn in 2D using tSNE. Cells are colored according to their corresponding cell type; the cell type of each simulated cell is based on the embedding parameter learned for the corresponding cell in the benchmark. Circles indicate cells from the benchmark, while triangles represent cells from the simulated dataset. Each plot illustrates the overlap of the same cell type across the benchmark and simulated datasets, while all other cell types are shown in grey. (**a**) HSPCs. (**b**) Progenitors. (**c**) Long term HSCs.


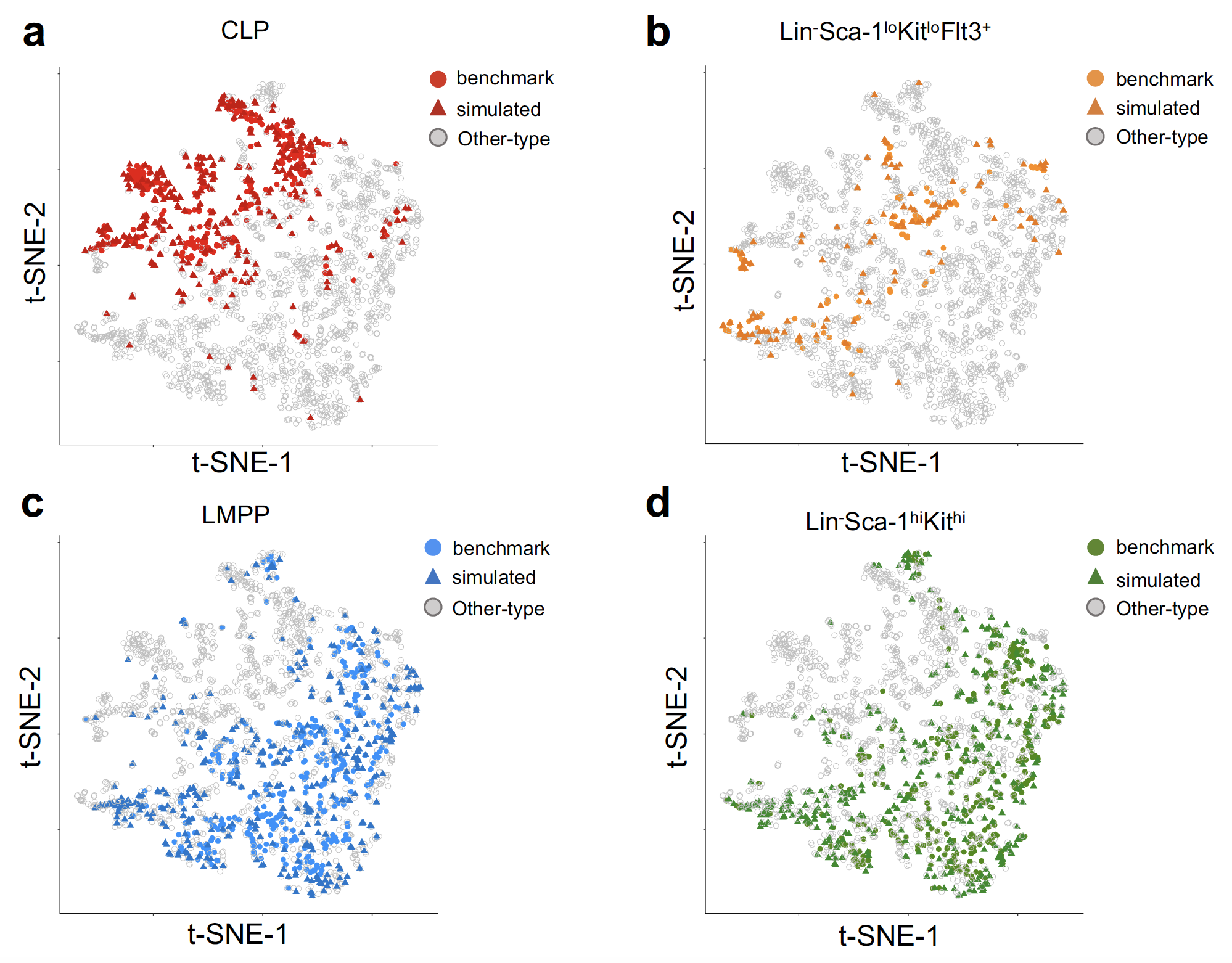


Fig S9: Simulation framework trained on the LSK benchmark generates simulated data that resemble the benchmark data. The simulation framework was used to generate a simulated dataset of the same size as the LSK benchmark with HVG selection. The combined dataset (simulated and benchmark) were jointly projected into five dimensions via PCA, then drawn in 2D using tSNE. Cells are colored according to their corresponding cell type; the cell type of each simulated cell is based on the embedding parameter learned for the corresponding cell in the benchmark. Circles indicate cells from the benchmark, while triangles represent cells from the simulated dataset. Each plot illustrates the overlap of the same cell type across the benchmark and simulated datasets, while all other cell types are shown in grey. (**a**) Common lymphoid progenitors. (**b**) Lin^-^Sca-1^lo^Kit^lo^Flt3^+^ cells. (**c**) Lymphoid-primed multipotent progenitors. (**d**) Lin^-^Sca-1^hi^Kit^hi^ cells.


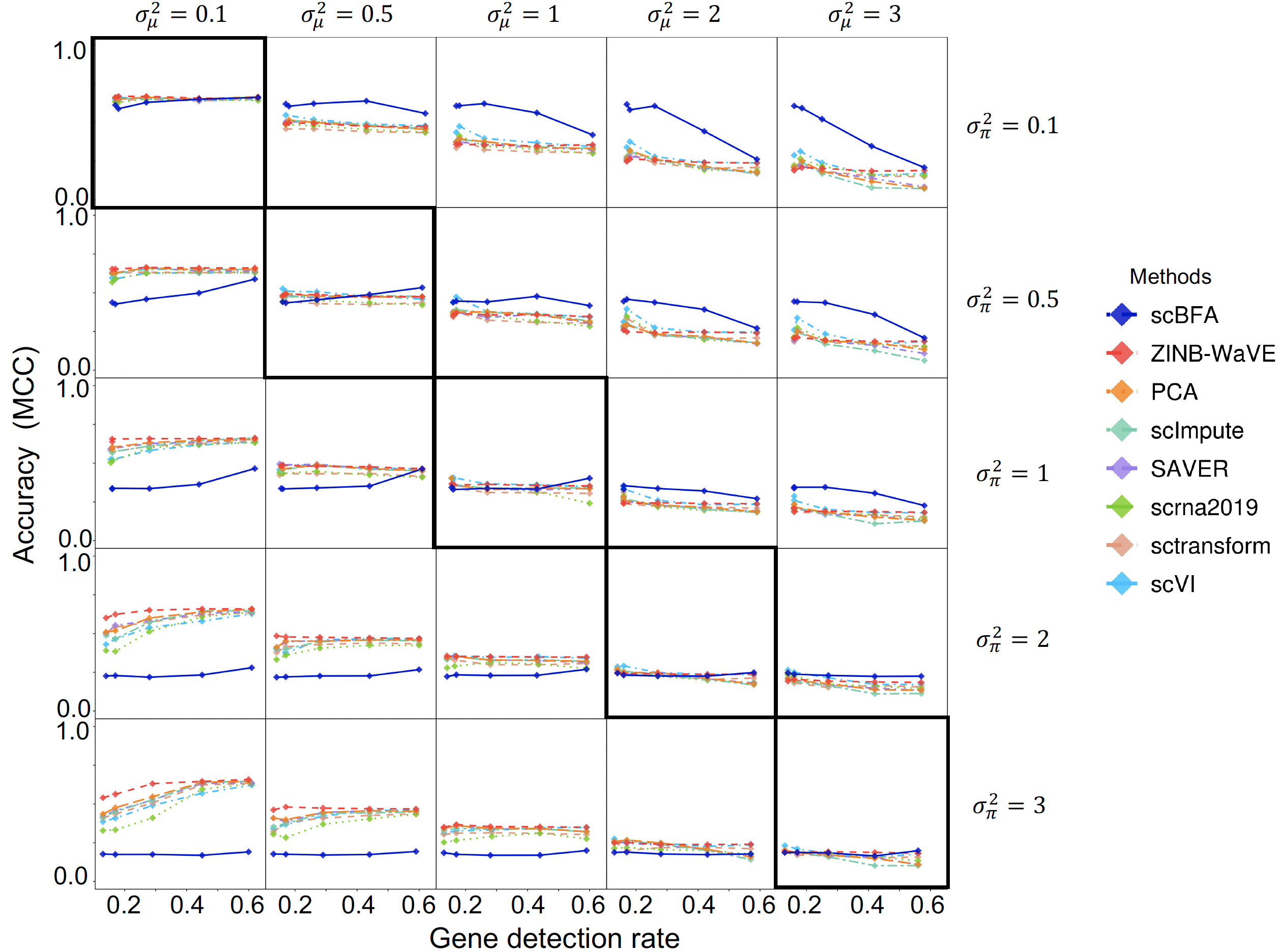


Fig S10: scBFA outperforms quantification models when the detection noise is smaller than the quantification noise (HVG, $r=5$). Rows represent different settings of (gene) detection noise ($\sigma_{\boldsymbol{\pi}}^{2}$), and columns represent different settings of (gene) quantification noise ($\sigma_{\mu}^{2}$). The diagonal represents simulations where the detection noise is equal to the quantification noise ($\sigma_{\mu}^{2}= \sigma_{\pi}^{2}$), and the plots above the diagonal represent simulations where the detection noise is less than the quantification noise. Each y-axis indicates the cross-validation performance (MCC) of cell type predictors trained on embeddings learned from the simulated data, while each x-axis represents the gene detection rate that is manipulated by the parameter $\delta$. Here, the ground-truth embedding matrix is obtained by fitting ZINB-WaVE to the LPS benchmark under HVG selection. The dispersion parameter $r$ is set to be 5 in these simulations.


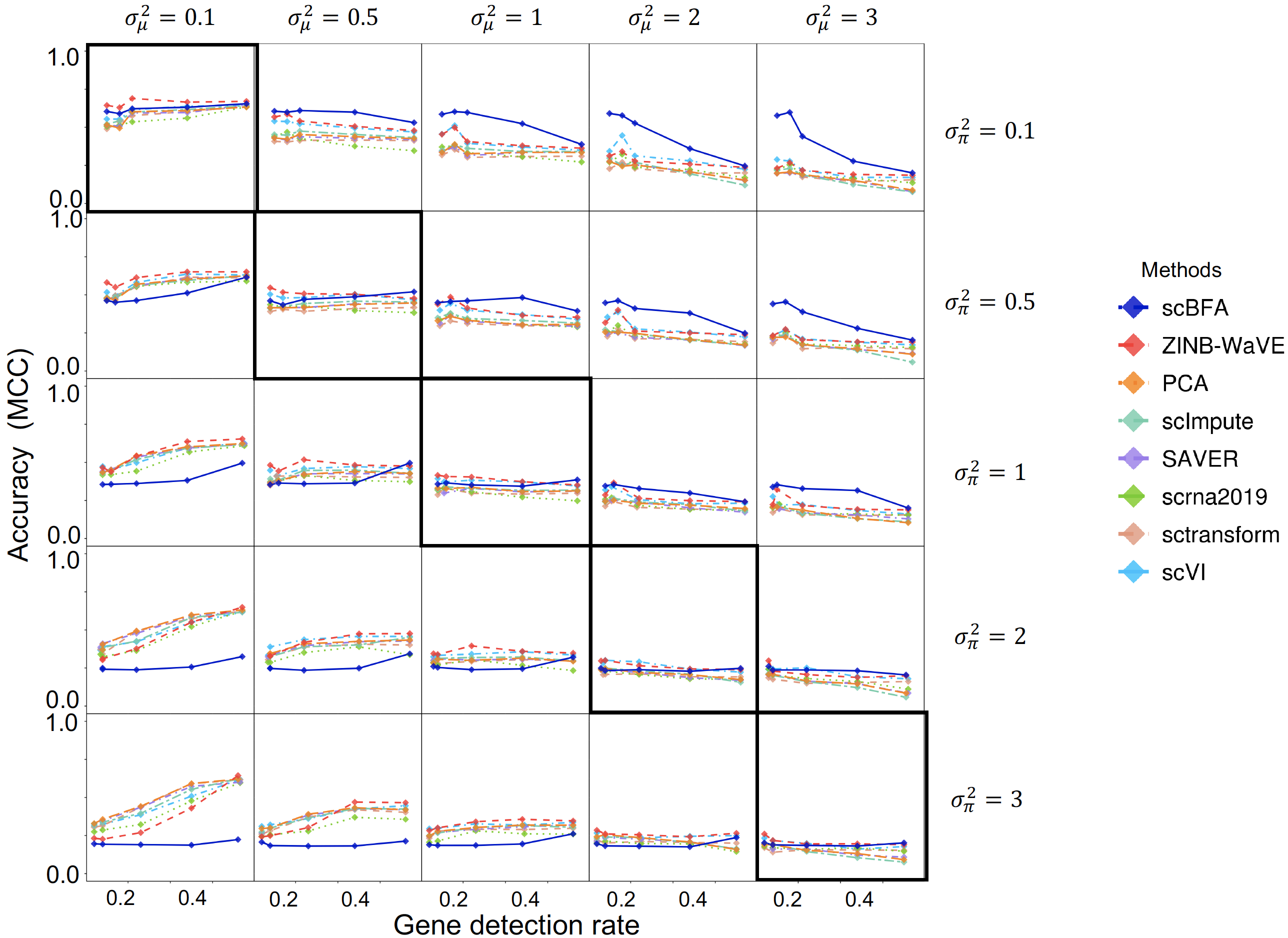


Fig S11: scBFA outperforms quantification models when the detection noise is smaller than the quantification noise (HVG, $r=0.5$). Rows represent different settings of (gene) detection noise ($\sigma_{\boldsymbol{\pi}}^{2}$), and columns represent different settings of (gene) quantification noise ($\sigma_{\mu}^{2}$). The diagonal represents simulations where the detection noise is equal to the quantification noise ($\sigma_{\mu}^{2}= \sigma_{\pi}^{2}$), and the plots above the diagonal represent simulations where the detection noise is less than the quantification noise. Each y-axis indicates the cross-validation performance (MCC) of cell type predictors trained on embeddings learned from the simulated data, while each x-axis represents the gene detection rate that is manipulated by the parameter $\delta$. Here, the ground-truth embedding matrix is obtained by fitting ZINB-WaVE to the LPS benchmark under HVG selection. The dispersion parameter $r$ is set to be 0.5 in these simulations.


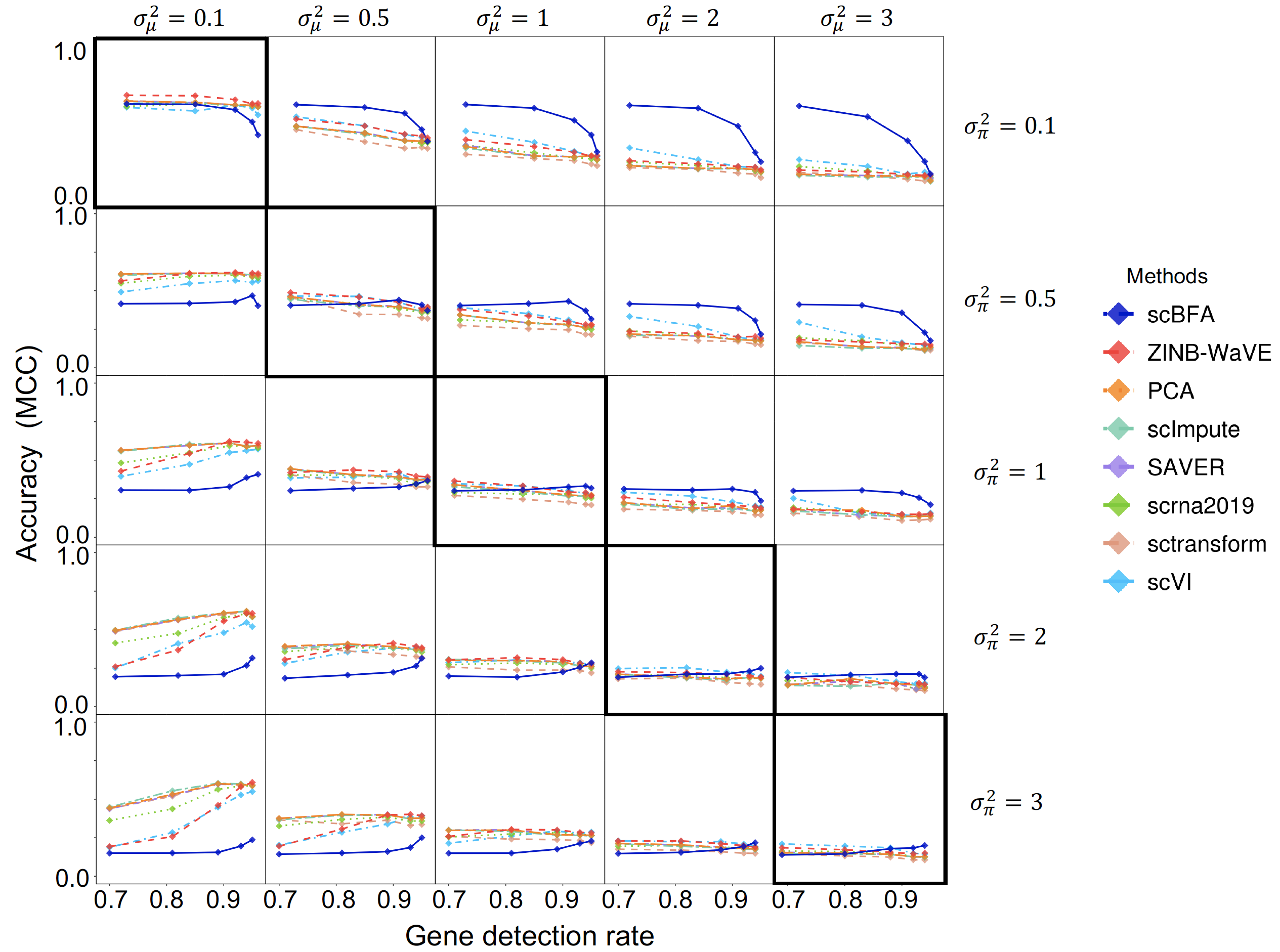


Fig S12: scBFA outperforms quantification models when gene detection noise is smaller than quantification noise (HEG, $r=1$). Rows represent different settings of (gene) detection noise ($\sigma_{\boldsymbol{\pi}}^{2}$), and columns represent different settings of (gene) quantification noise ($\sigma_{\mu}^{2}$). The diagonal represents simulations where the detection noise is equal to the quantification noise ($\sigma_{\mu}^{2}= \sigma_{\pi}^{2}$), and the plots above the diagonal represent simulations where the detection noise is less than the quantification noise. Each y-axis indicates the cross-validation performance (MCC) of cell type predictors trained on embeddings learned from the simulated data, while each x-axis represents the gene detection rate that is manipulated by the parameter $\delta$. Here, the ground-truth embedding matrix is obtained by fitting ZINB-WaVE to the LPS benchmark under HEG selection. The dispersion parameter $r$ is set to be 1 in these simulations.


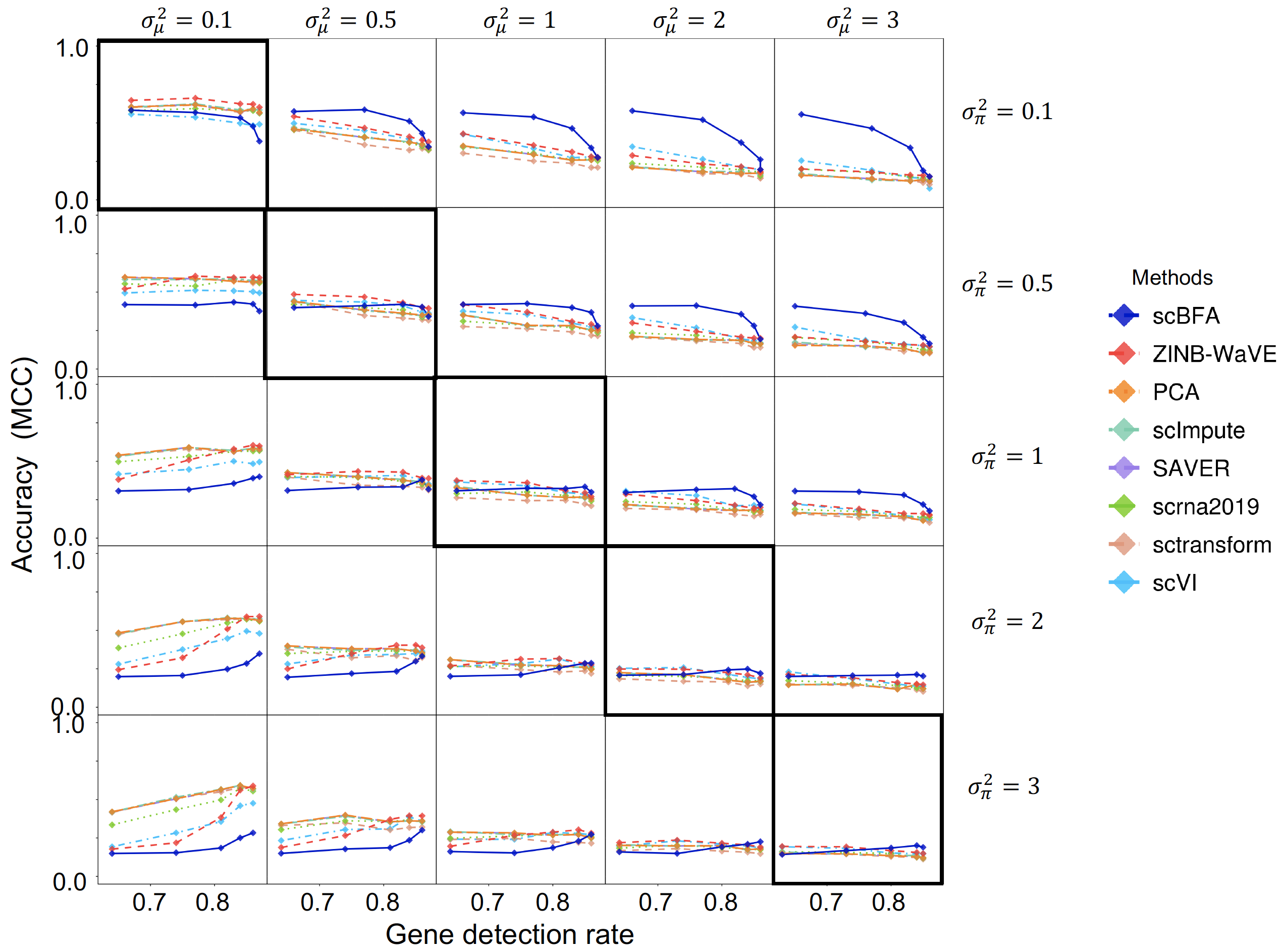


Fig S13: scBFA outperforms quantification models when gene detection noise is smaller than quantification noise (HEG, $r=0.5$). Rows represent different settings of (gene) detection noise ($\sigma_{\boldsymbol{\pi}}^{2}$), and columns represent different settings of (gene) quantification noise ($\sigma_{\mu}^{2}$). The diagonal represents simulations where the detection noise is equal to the quantification noise ($\sigma_{\mu}^{2}= \sigma_{\pi}^{2}$), and the plots above the diagonal represent simulations where the detection noise is less than the quantification noise. Each y-axis indicates the cross-validation performance (MCC) of cell type predictors trained on embeddings learned from the simulated data, while each x-axis represents the gene detection rate that is manipulated by the parameter $\delta$. Here, the ground-truth embedding matrix is obtained by fitting ZINB-WaVE to the LPS benchmark under HEG selection. The dispersion parameter $r$ is set to be 0.5 in these simulations.


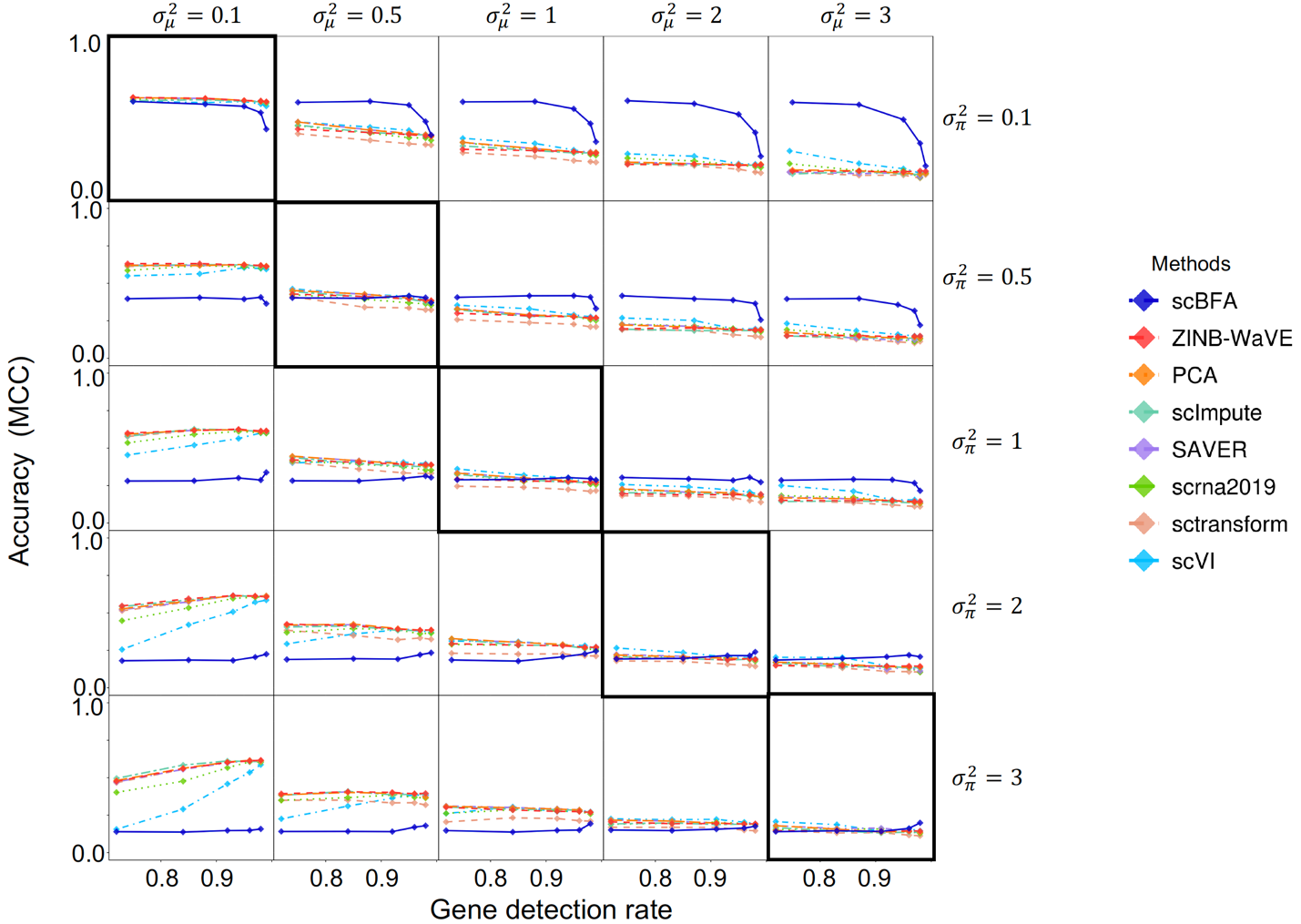


Fig S14: scBFA outperforms quantification models when gene detection noise is smaller than quantification noise (HEG, $r=5$). Rows represent different settings of (gene) detection noise ($\sigma_{\boldsymbol{\pi}}^{2}$), and columns represent different settings of (gene) quantification noise ($\sigma_{\mu}^{2}$). The diagonal represents simulations where the detection noise is equal to the quantification noise ($\sigma_{\mu}^{2}= \sigma_{\pi}^{2}$), and the plots above the diagonal represent simulations where the detection noise is less than the quantification noise. Each y-axis indicates the cross-validation performance (MCC) of cell type predictors trained on embeddings learned from the simulated data, while each x-axis represents the gene detection rate that is manipulated by the parameter $\delta$. Here, the ground-truth embedding matrix is obtained by fitting ZINB-WaVE to the LPS benchmark under HEG selection. The dispersion parameter $r$ is set to be 5 in these simulations.


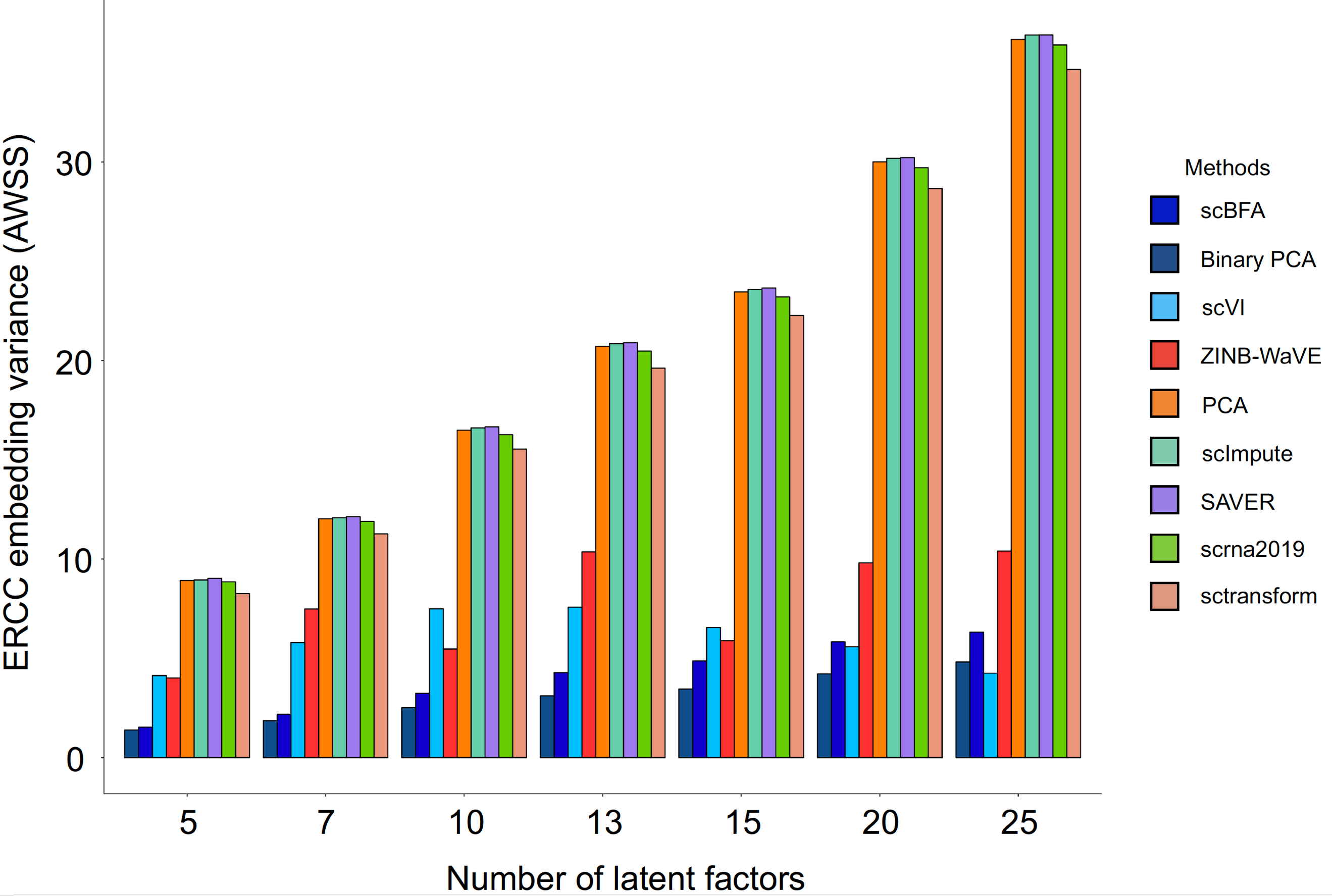


Fig S15: Comparison of the variance in embedding dimensions of the ERCC dataset **of Zheng et al.** Methods are compared based on the variance of the embeddings learned over the ERCC dataset generated by Zheng et al. Variance is measured as AWSS (Average Within-Group Sum of Squares), as a function of the number of latent dimensions specified. Gene detection models (scBFA and Binary PCA) yield embeddings with lowest variance.

Fig S16: Distribution of the fraction of reads of each cell mapping to mitochondrial genes in the Dendritic benchmark of Shalek et al. The fraction is calculated based on the HEG selection criterion.


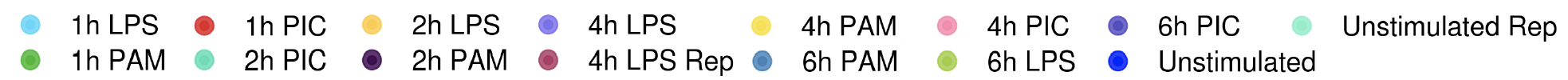


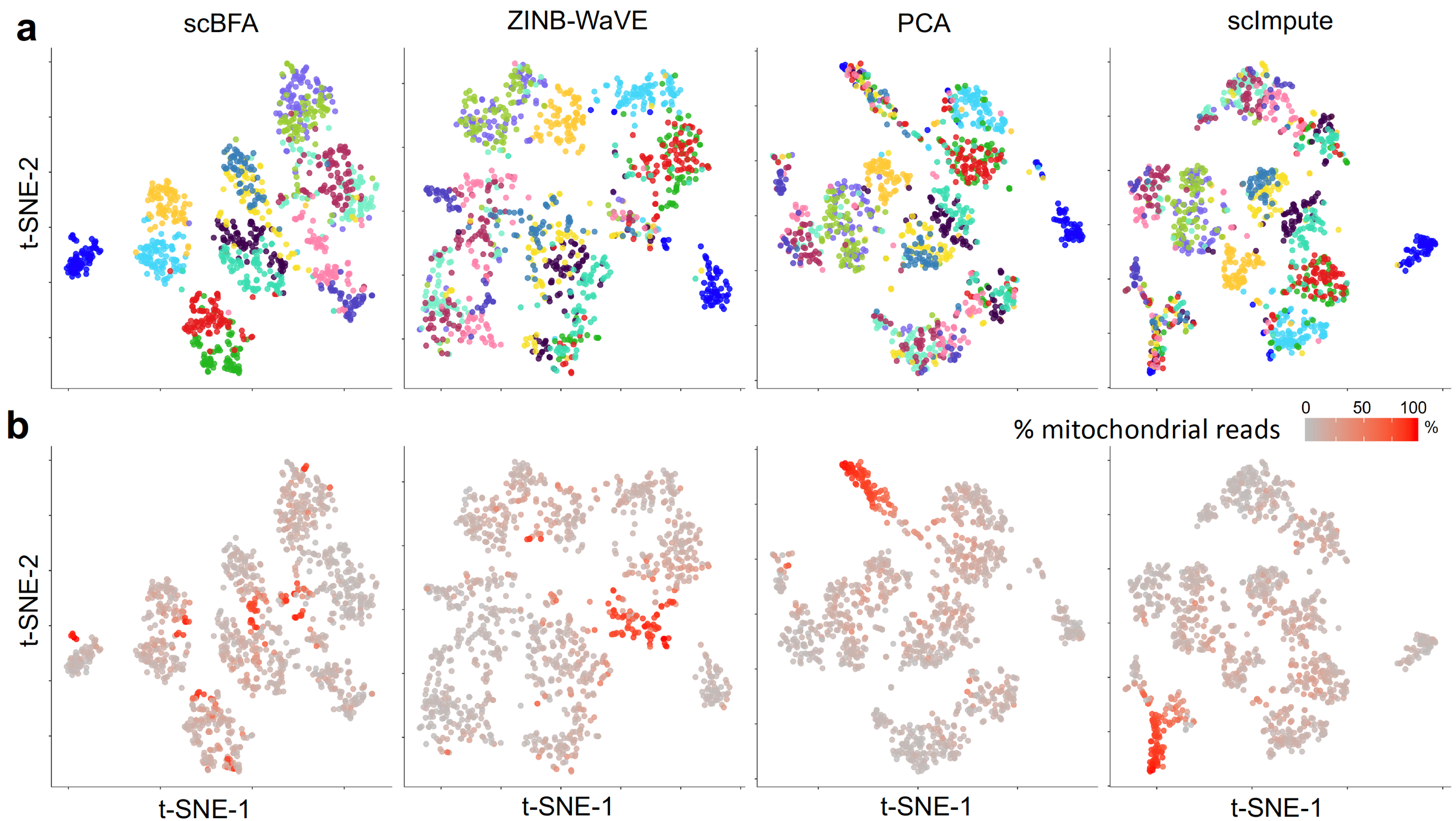


Fig S17: 2D tSNE visualization of the Dendritic benchmark of Shalek et al. (**a**) 2D tSNE visualization of the 10-dimensional embedding generated by scBFA, ZINB-WaVE, PCA and scImpute on the Dendritic benchmark under HEG selection, when cells with high mitochondrial RNA content are kept in the analysis. Cells are colored according to their corresponding cell types and states**.** (**b**) Same visualization as in (a), but cells are colored according to the fraction of their reads that map to mitochondrial genes.


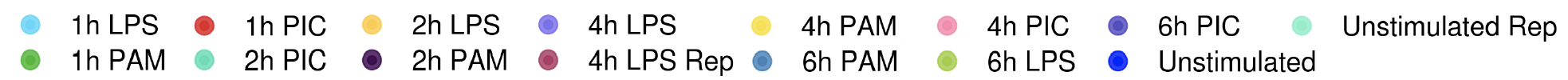


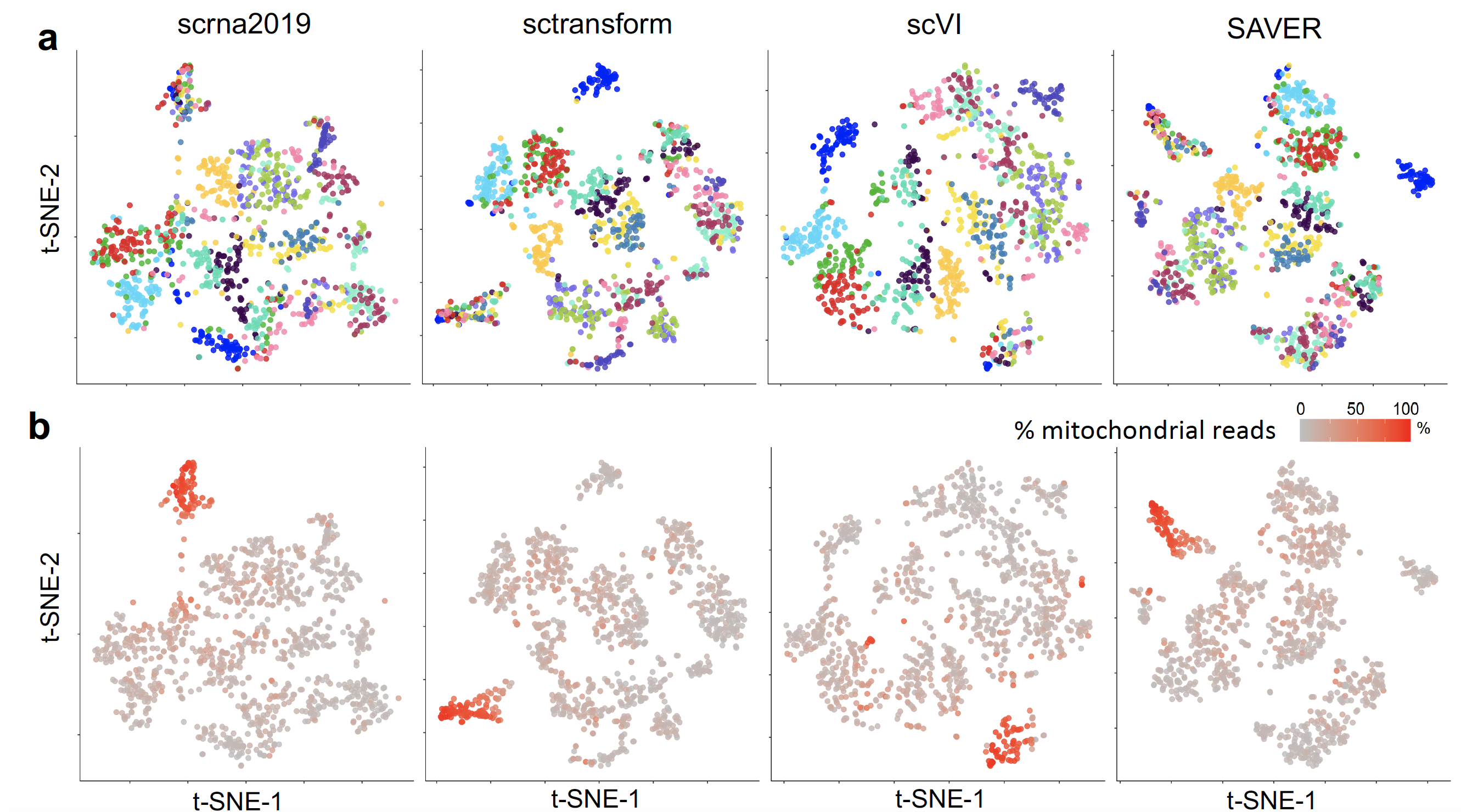


Fig S18: 2D tSNE visualization of the Dendritic benchmark of Shalek et al. (**a**) 2D tSNE visualization of the 10-dimensional embedding generated by scrna2019, sctransform, scVI and SAVER on the Dendritic benchmark under HEG selection, when cells with high mitochondrial RNA content are kept in the analysis. Cells are colored according to their corresponding cell types and states**.** (**b**) Same visualization as in (a), but cells are colored according to the fraction of their reads that map to mitochondrial genes.


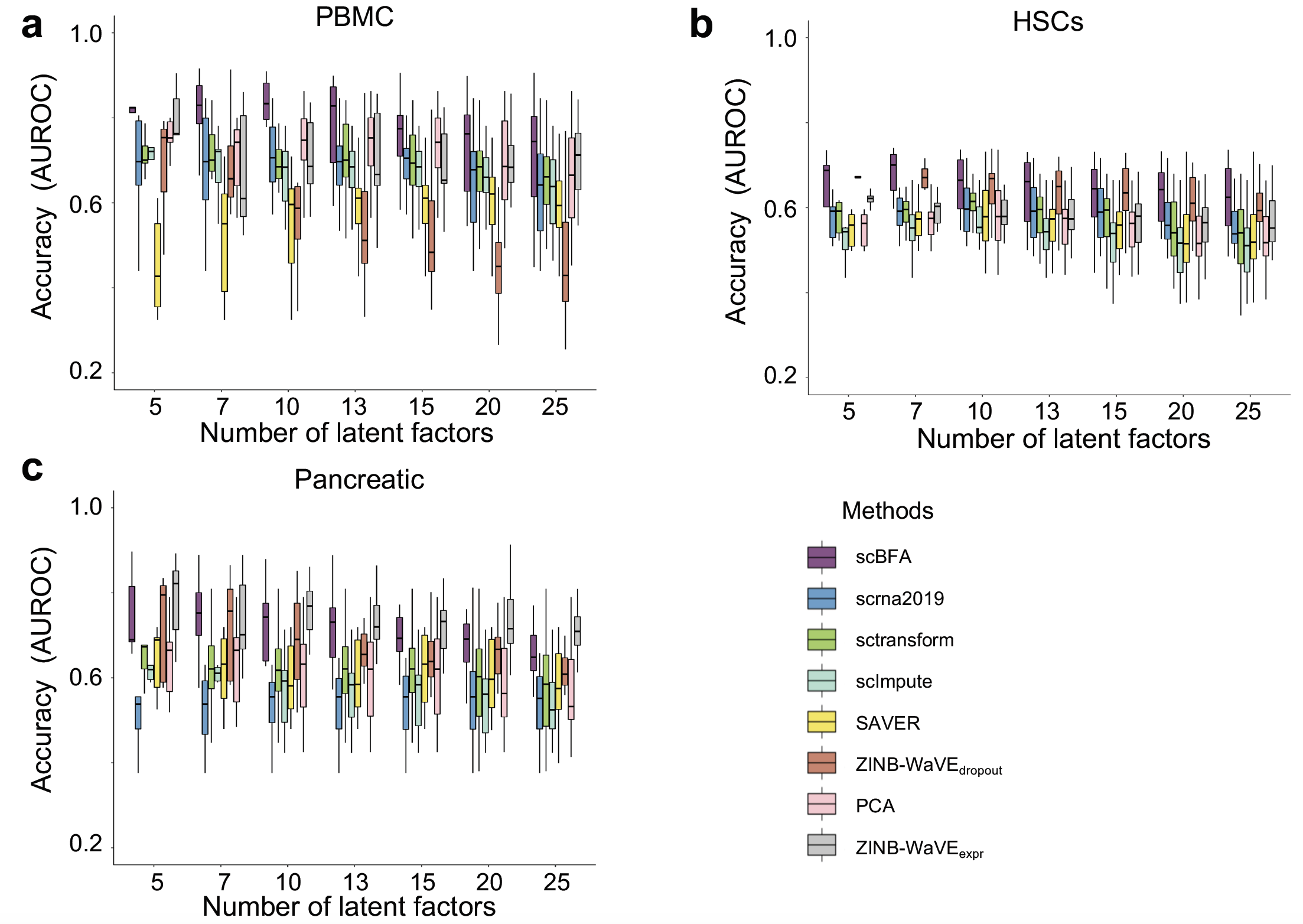


Fig S19: scBFA is better informed by cell type markers than quantification models, under HEG selection. Each latent factor learned from each method was evaluated based on how much influence established cell type markers had on its embeddings, as measured by the area under the curve (AUROC) metric. Each boxplot represents the AUROC of all latent factors for a given method, for a given benchmark. ZINB-WaVE is represented twice, once for the latent dimensions inferred by their gene detection pattern (ZINB-WaVE_dropout_), and once for the latent dimensions inferred from the gene counts (ZINB-WaVE_expr_). (**a**) PBMC benchmark. (**b**) HSCs benchmark. (**c**) Pancreatic benchmark.


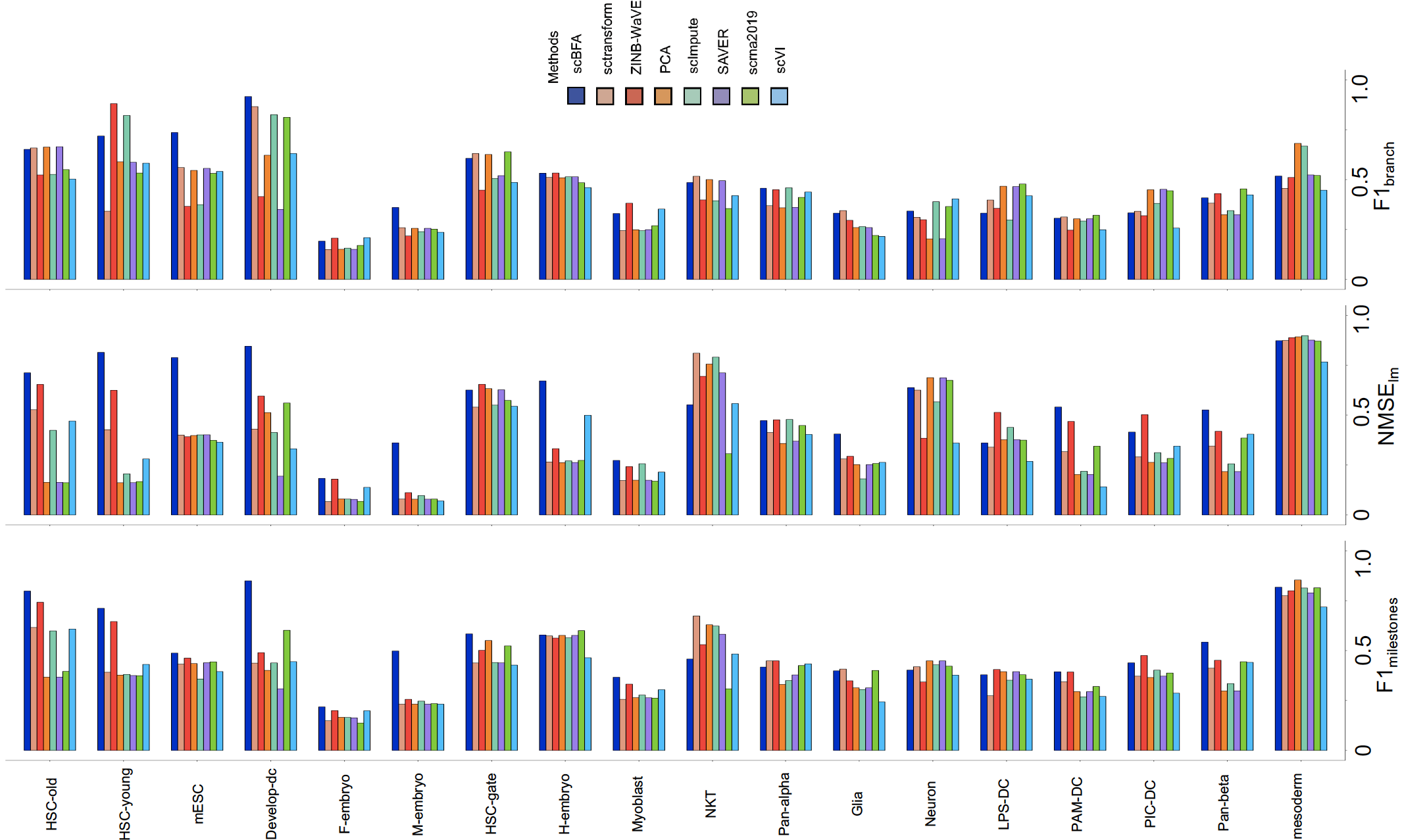


Fig S20: scBFA improves trajectory inference of the method Slingshot compared to other dimensionality reduction methods. The y-axis represents the set of 18 “gold standard” trajectory inference benchmarks from Dynverse. These benchmarks are processed under HVG selection. For each dimensionality reduction method, the trajectory inference method Slingshot was adapted by replacing its PCA step with a corresponding dimensionality reduction method, and the resulting performance evaluated. Performance here is measured via the F1_milestone_, NMSE_lm_ and F1_branch_ scores that measure how well the inferred trajectory matches the ground truth provided by Dynverse. F1_milestone_ and F1_branch_ are based on the quality of clustering of cells in the trajectory, while NMSE_lm_ assesses how well the position of a cell in the inferred trajectory predicts the position of the cell in the ground truth trajectory.


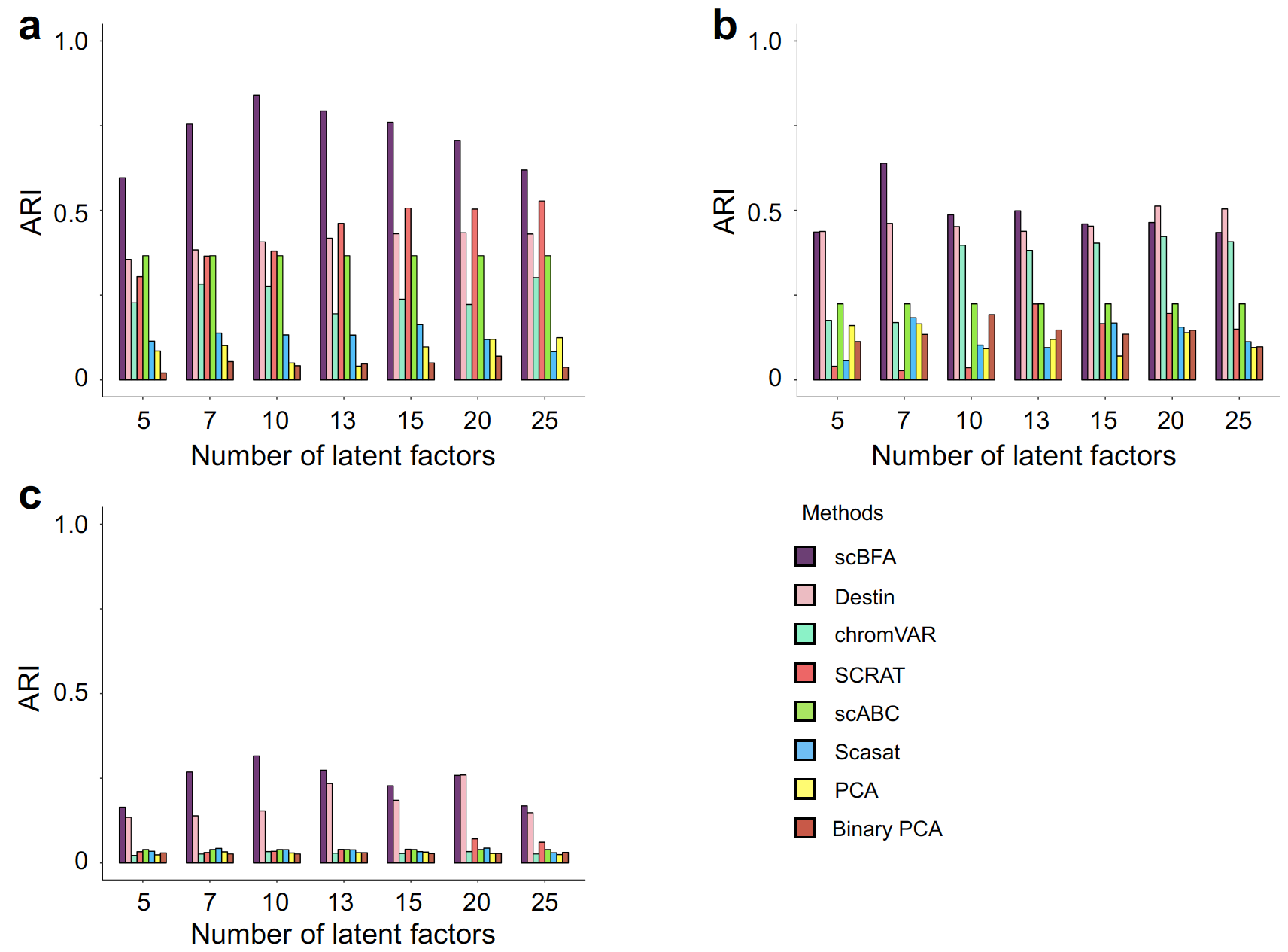


Fig S21: scBFA accurately recovers cell type identity in scATAC-seq benchmarks. ARI (Adjusted Rand Index) measures the clustering accuracy of different methods on each scATAC-seq benchmark, as a function of the number of latent dimensions specified. The benchmarks from left to right, top to bottom are GSE96769, GSE74310 and GSE107816.


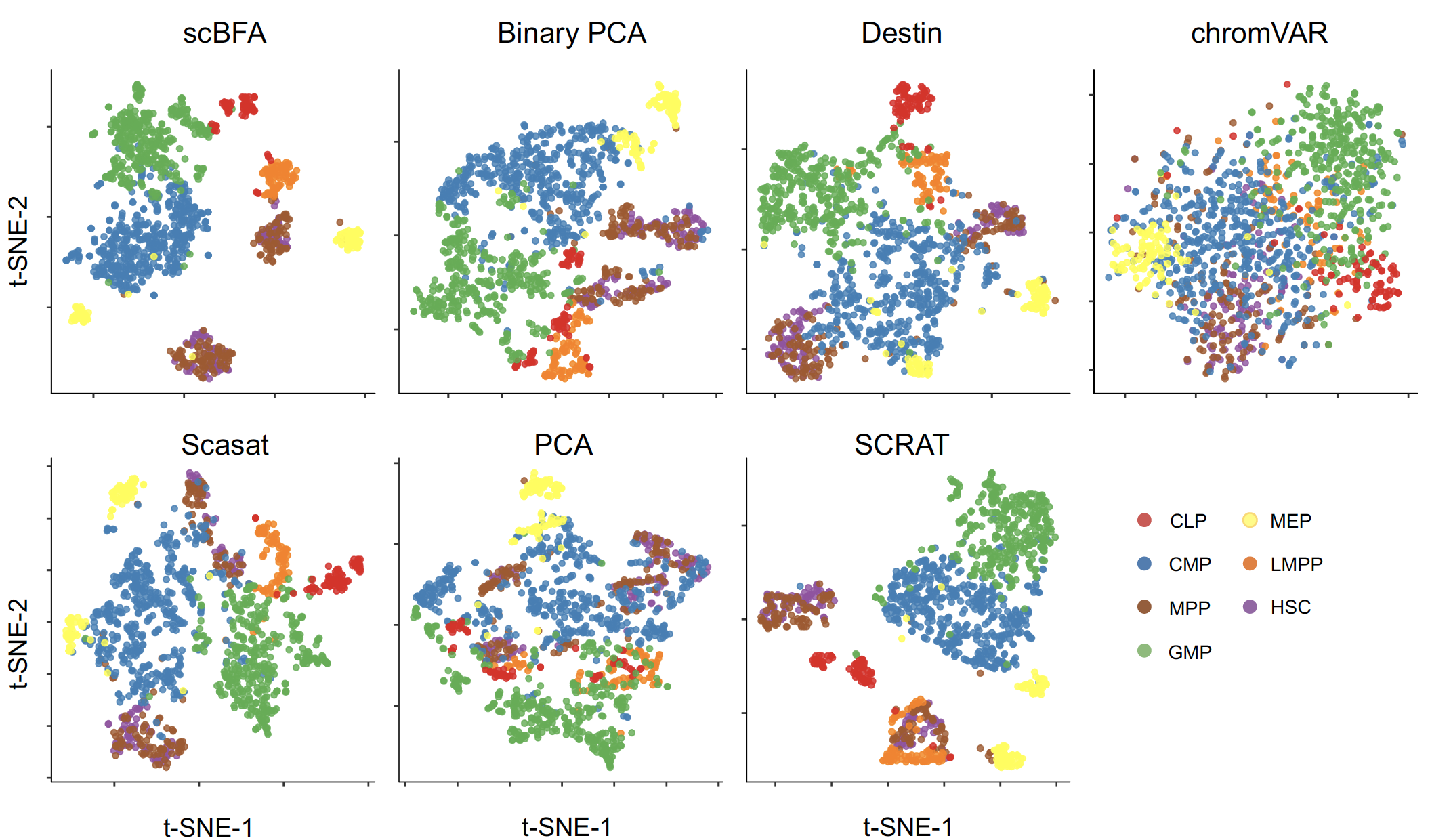


Fig S22: 2D tSNE visualization of the scATAC-seq benchmark GSE96769. tSNE plots are generated based on the 10-dimensional embeddings learned by scBFA, Scasat, Binary PCA, PCA, Destin, chromVAR and SCRAT. Cells are colored by their corresponding cell types.


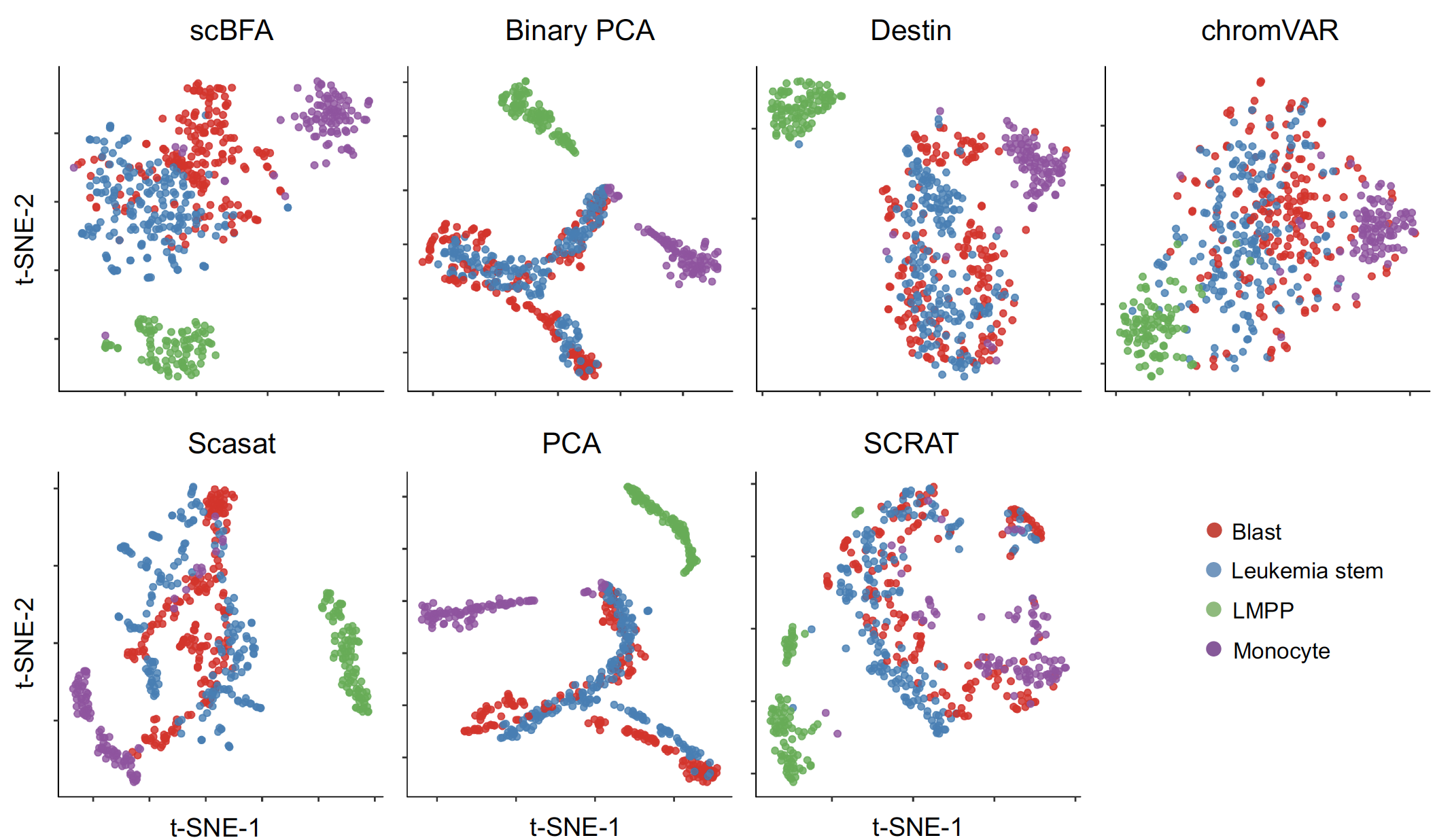


Fig S23: 2D tSNE visualization of the scATAC-seq benchmark GSE74310. tSNE plots are generated based on the 10-dimensional embeddings learned by scBFA, Scasat, Binary PCA, PCA, Destin, chromVAR and SCRAT. Cells are colored by their corresponding cell types.


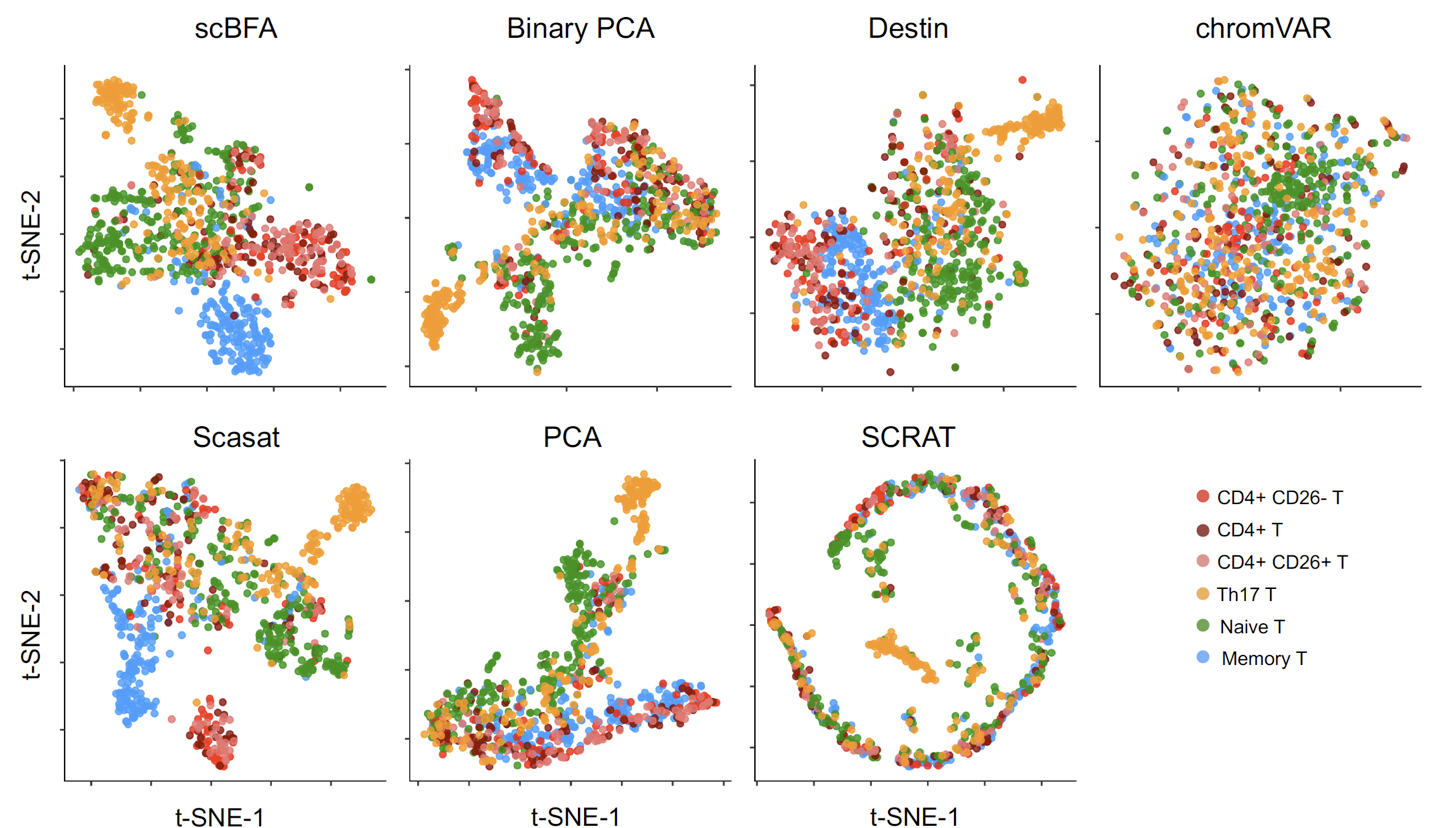


Fig S24: 2D tSNE visualization of the scATAC-seq benchmark GSE107816. tSNE plots are generated based on the 10-dimensional embeddings learned by scBFA, Scasat, Binary PCA, PCA, Destin, chromVAR and SCRAT. Cells are colored by their corresponding cell types.


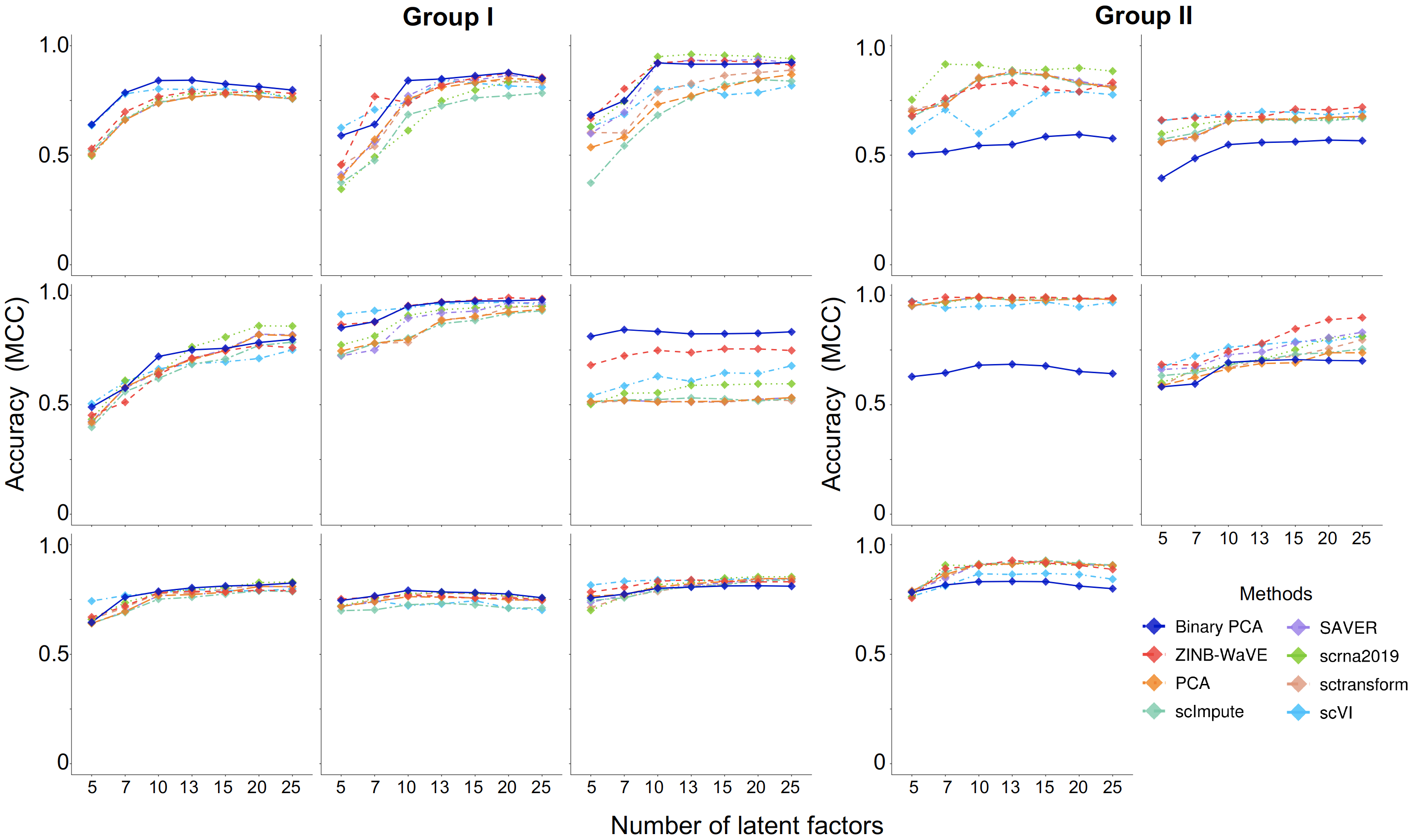


Fig S25: Binary PCA performance versus quantification models under HEG selection. Performance is measured via cross-validation of cell type classifiers trained on scRNA-seq benchmark data in the respective embedding spaces of each method, as a function of the number of latent dimensions specified. Benchmarks are grouped based on whether scBFA is a top performer (Group I) or performs poorly (Group II). The set of Group I benchmarks from left to right, top to bottom: Dendritic, Pancreatic, DC, MGE, Intestinal, MEM-T, Myeloid, HSCs, PBMC. The set of Group II benchmarks from left to right: mESCs, HSPC, H7-ESC, LSK and LPS.


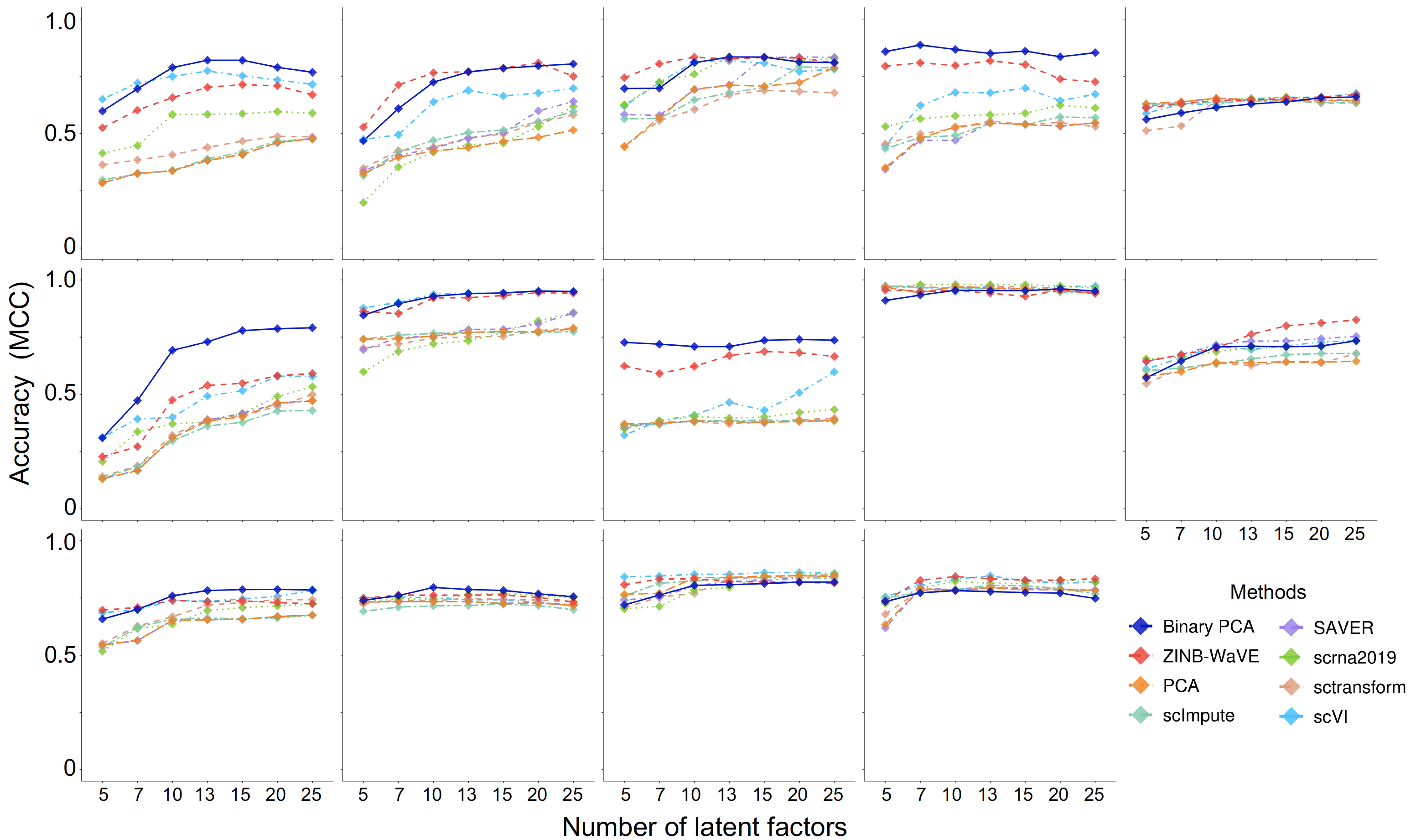


Fig S26: Binary PCA performance versus quantification models under HVG selection. Cross-validation performance of cell type classifiers trained on low dimensional embeddings of scRNA-seq benchmark data, as a function of the number of latent dimensions specified. Benchmarks from left to right, top to bottom: Dendritic, Pancreatic, DC, mESCs, HSPC, MGE, Intestinal, MEM-T, H7-ESC, LSK, Myeloid, HSCs, PBMC, and LPS.

**
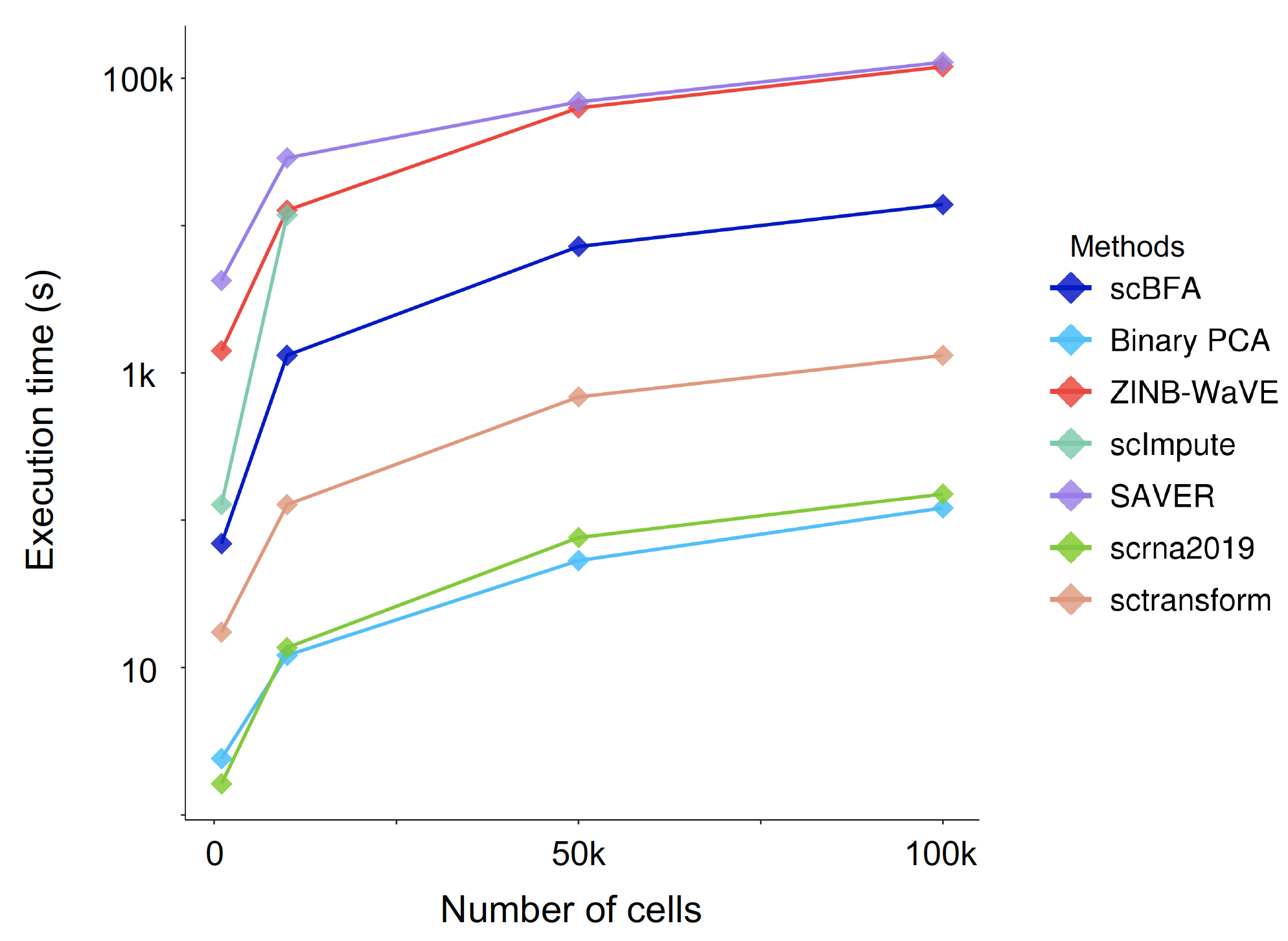
**

Fig S27: Fast scBFA approximation is one of the fastest dimensionality reduction methods. Execution time of gene detection models (scBFA and Binary PCA) versus quantification models (ZINB-WaVE, scImpute, scrna2019, sctransform, SAVER) on different dataset sizes, as subsampled from the 1.3 million scRNA-seq mouse brain dataset generated from 10x Genomics.

Fig S28: Schematic of scBFA. $B$ is a matrix of size $N$ (number of cells) by $G$ (number of genes) encoding the gene detection pattern of the input data, where $B_{ij}=0$ if no molecule (UMI or read) maps to gene $i$ in cell $j$, otherwise $B_{ij}=1$. BFA decomposes $B$ into a $K$-dimensional embedding matrix $Z$ (factor scores) and a loading matrix $A$. An optional observed cell covariate matrix $X$ is used to model batch effects and other nuisance cell-wise factors. $\beta$ is the corresponding coefficient matrix of $X$.


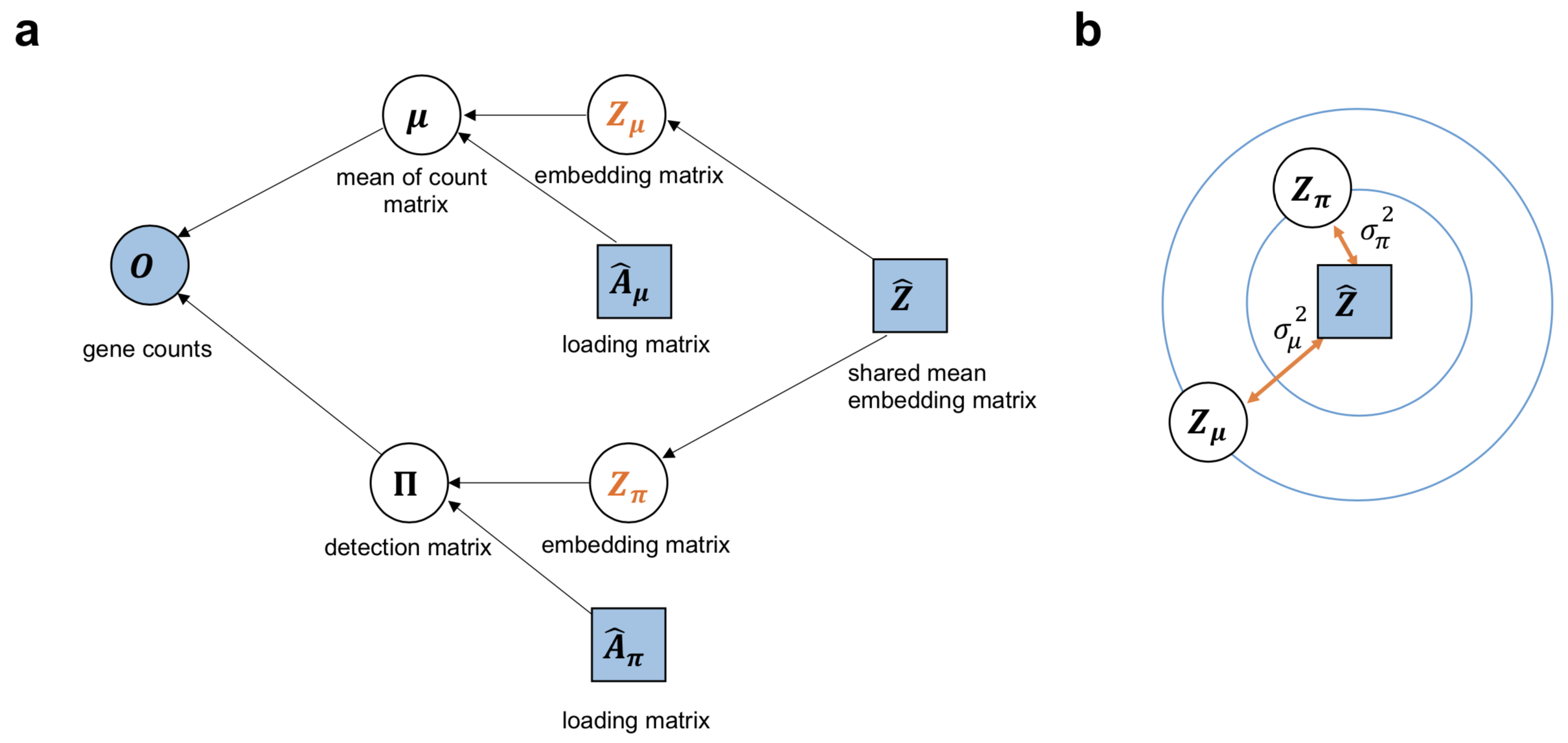


Fig S29: The main components of the generative process of the scRNA-seq simulation framework**.** (**a**) Given $N$ cells ($i=1,\ldots,N)$and $G$ genes $\left( j=1,\ldots,G \right)$, our simulation ultimately seeks to generate an observed gene count matrix $\boldsymbol{O}$**.** $\boldsymbol{O}$ depends on two internal latent variables: a matrix $\boldsymbol{\mu}$ representing gene counts, and a matrix $\boldsymbol{\Pi}$ representing the gene detection pattern**.** If $\boldsymbol{\Pi}_{\boldsymbol{i}j}=1$, then ${\boldsymbol{O}_{ij}}=0$. Otherwise, if $\boldsymbol{\Pi}_{\boldsymbol{i}j}=0$, then $\boldsymbol{O}_{ij}$ is sampled from a negative binomial distribution whose mean is $\mu_{ij}$. $\boldsymbol{\mu}$ is determined by the product of a count-based embedding space $\boldsymbol{Z}_{\boldsymbol{\mu}}$ and loading matrix ${\hat{\boldsymbol{A}}}_{\boldsymbol{\mu}}$**,** while $\boldsymbol{\Pi}$ is sampled from a Bernoulli distribution that is parameterized through the product of a separate detection-based embedding $\boldsymbol{Z}_{\boldsymbol{\pi}}$ and loading ${\hat{\boldsymbol{A}}}_{\boldsymbol{\pi}}$**.** ${\hat{\boldsymbol{A}}}_{\boldsymbol{\mu}}$ and ${\hat{\boldsymbol{A}}}_{\boldsymbol{\pi}}$ are parameters that are estimated prior to the simulation by applying ZINB-WaVE to a real dataset. The count and detection-based embeddings $\boldsymbol{Z}_{\boldsymbol{\mu}}$ and $\boldsymbol{Z}_{\boldsymbol{\pi}}$ respectively are sampled from a distribution with the same mean $\hat{\boldsymbol{Z}}$ (also learned from applying ZINB-WaVE to real data) but with separate variance terms $\sigma_{\mu}^{2}$ and $\sigma_{\pi}^{2}$, respectively. These separate variance terms allow us to simulate different noise levels for detection and quantification. (**b**) Graphical illustration indicating that $\boldsymbol{Z}_{\boldsymbol{\mu}}$ and $\boldsymbol{Z}_{\boldsymbol{\pi}}$ are sampled from a common distribution centered on $\hat{\boldsymbol{Z}}$, though with separate variance terms $\sigma_{\mu}^{2}$ and $\sigma_{\pi}^{2}$, respectively.

# Table S1: Summary of scRNA-seq cell type identification benchmark datasets.

| **Dataset** | **# of cell types** | **ID** | **# of cells** | **Year** | **Protocol** | **Source** |
| --- | --- | --- | --- | --- | --- | --- |
| Dendritic | 15 | GSE48968 | 1378 | 2014 | full length | <http://imlspenticton.uzh.ch/robinson_lab/conquer/data-mae/GSE48968-GPL13112.rds> |
| MGE | 5 | GSE104157 | 1124 | 2017 | full length | <https://www.ncbi.nlm.nih.gov/geo/query/acc.cgi?acc=GSE104157> |
| HSCs | 6 | GSE100426 | 949 | 2017 | full length | <https://www.ncbi.nlm.nih.gov/geo/query/acc.cgi?acc=GSE100426> |
| Intestinal | 4 | GSE62270 | 2891 | 2015 | UMI | <http://imlspenticton.uzh.ch/robinson_lab/conquer/data-mae/GSE62270-GPL17021.rds> |
| MEM-T | 7 | GSE106540 | 2244 | 2018 | UMI | <https://www.ncbi.nlm.nih.gov/geo/query/acc.cgi?acc=GSE106540> |
| Pancreatic | 5 | GSE81076 | 1152 | 2016 | UMI | <http://imlspenticton.uzh.ch/robinson_lab/conquer/data-mae/GSE81076-GPL18573.rds> |
| PBMC | 10 | GSE100866 | 7666 | 2017 | UMI | <https://www.ncbi.nlm.nih.gov/geo/query/acc.cgi?acc=GSE100866> |
| DC | 4 | GSE89232 | 950 | 2016 | full length | <https://www.ncbi.nlm.nih.gov/geo/query/acc.cgi?acc=GSE89232> |
| Myeloid | 5 | GSE123025 | 1816 | 2018 | full length | <https://www.ncbi.nlm.nih.gov/geo/query/acc.cgi?acc=GSE123025> |
| H7-ESC | 9 | SRP073808 | 651 | 2016 | full length | <http://imlspenticton.uzh.ch/robinson_lab/conquer/data-mae/SRP073808.rds> |
| mESCs | 3 | EMTAB2805 | 288 | 2015 | full length | <http://imlspenticton.uzh.ch/robinson_lab/conquer/data-mae/EMTAB2805.rds> |
| LPS | 5 | GSE94383 | 839 | 2017 | full length | <http://imlspenticton.uzh.ch/robinson_lab/conquer/data-mae/GSE94383.rds> |
| LSK | 4 | GSE100037 | 1188 | 2017 | UMI | <https://www.ncbi.nlm.nih.gov/geo/query/acc.cgi?acc=GSE100037> |
| HSPC | 3 | GSE81682 | 1826 | 2016 | full length | <https://www.ncbi.nlm.nih.gov/geo/query/acc.cgi?acc=GSE81682> |
|  |  |  |  |  |  |  |

# Table S2: Summary of gene intersection between the top 2,000 genes selected by HVG and HEG

| **Dataset** | **# genes in intersection between HVG and HEG** |
| --- | --- |
| Dendritic | 345 |
| MGE | 293 |
| HSCs | 906 |
| Intestinal | 490 |
| MEM-T | 383 |
| Pancreatic | 691 |
| PBMC | 582 |
| DC | 301 |
| Myeloid | 398 |
| H7-ESC | 185 |
| mESCs | 227 |
| LPS | 260 |
| LSK | 613 |
| HSPC | 60 |
|  |  |

# Table S3: Cell surface marker list for the PBMC benchmark.

| **Marker Genes** | **Source** |
| --- | --- |
| CD3D, CD8A, NKG7, FCER1A, CD16, S100A8, S100A9, CD79A, CD79b, CD4, CCR10, PF4, TNFRSF18 | <https://www.nature.com/articles/ncomms14049> |
| CD94, GATA3 (Important regulator), NKG2 | <https://link.springer.com/content/pdf/10.1385%2FIR%3A35%3A3%3A263.pdf> |
| CD163, GZMB, FCRL2, CD40LG, CRTAM, SIGLEC10, FOXP3, IL17A, CXCR5, KLRC1, PRF1, LINC00926, RP11-291B21.2 | <https://www.ncbi.nlm.nih.gov/pmc/articles/PMC1895851/> |
| GZMB, HLA_DPB1, DUSP5, LY96, NOD2, STAM, CTSW, CD160, CD244, NCR3 | <http://journals.plos.org/plosbiology/article?id=10.1371/journal.pbio.1001148> |
| CD3G, LEF1, TCF7 | <https://www.ncbi.nlm.nih.gov/pubmed/16424171> |
| CD8B | <https://www.ncbi.nlm.nih.gov/pmc/articles/PMC4686144/> |

# Table S4: Cell surface marker list for the HSC benchmark.

| **Marker Genes** | **Source** |
| --- | --- |
| Tnf, Irf1, Tlr2, Cxcl2, Traf1, Ccl5, Cxcl1,  Clec4e, Saa3, Il1a, Il1b, Mmp13, Il10, Lcn2, Il12b, Nos2, Ifitm3 | <https://www.ncbi.nlm.nih.gov/pubmed/30540934> |
| Gfi1b, Selp, Klf1, Pf4, Gp9, Zfpm1, Ikzf1, Ikzf2, Flt3, Il7ra | <http://www.bloodjournal.org/content/121/22/4463?sso-checked=true> |
| Tie2 | <https://www.ncbi.nlm.nih.gov/pubmed/15260986> |
| Alcam | <https://www.ncbi.nlm.nih.gov/pubmed/23280653> |
| Kit | <http://jem.rupress.org/content/211/2/217> |
| Slamf1 | <https://www.ncbi.nlm.nih.gov/pmc/articles/PMC2851806/> |
| Matk/Chk | <https://www.ncbi.nlm.nih.gov/pmc/articles/PMC1895851/> |
| Cd81 | <http://journals.plos.org/plosbiology/article?id=10.1371/journal.pbio.1001148> |
| Ccl5 | <http://www.bloodjournal.org/content/bloodjournal/119/11/2500.full.pdf?sso-checked=true> |
| Runx1, Notch1, Meis1 | <https://link.springer.com/chapter/10.1007%2F978-94-007-6621-1_11> |
| Ly6a | <https://www.ncbi.nlm.nih.gov/pubmed/22136929> |
| Egr1 | <https://www.sciencedirect.com/science/article/pii/S1934590908000568> |
| Mecom | <https://www.ncbi.nlm.nih.gov/pubmed/21666053> |
| Vwf | <https://www.ncbi.nlm.nih.gov/pmc/articles/PMC3879699/> |
| Lmo2 | <https://www.ncbi.nlm.nih.gov/pmc/articles/PMC3092146> |
| Igta2b | <https://journals.plos.org/plosone/article?id=10.1371/journal.pone.0043300> |
| Flt3 | <http://journals.plos.org/plosone/article?id=10.1371/journal.pone.0138257> |
| Gata2 | <https://www.ncbi.nlm.nih.gov/pmc/articles/PMC4797020/> |
| Gfi1, Gfi1b | <https://www.ncbi.nlm.nih.gov/pmc/articles/PMC524350/> |
| Meis1 | <https://www.ncbi.nlm.nih.gov/pmc/articles/PMC4795694/> |
| Gata3 | <https://www.pnas.org/content/102/7/2448> |
| Ly6a | <https://www.ncbi.nlm.nih.gov/pubmed/22136929> |

# Table S5: Cell surface marker list for the Pancreatic benchmark.

| **Marker Genes** | **Source** |
| --- | --- |
| GCG, KRT19, PRSS1, SST, GCG, INS, PPY, FTH1, KRT7, PRSS2 | <https://www.cell.com/cell-stem-cell/fulltext/S1934-5909(16)30094-7?_returnURL=https%3A%2F%2Flinkinghub.elsevier.com%2Fretrieve%2Fpii%2FS1934590916300947%3Fshowall%3Dtrue> |
| IRX2, GC, LOXL4, SPP1, IAPP, PNLIP, PRG4, IAPP, ALDH1A1, PCSK2 | <https://www.cell.com/cell-systems/pdf/S2405-4712(16)30292-7.pdf> |
| TSPAN7, FXYD2, TMEM27, SEZ6L2, LRP11, DISP2, DDR1, DNER, NPTX2 | <https://link.springer.com/article/10.1007%2Fs00125-011-2295-1> |
| SCG5, GAD2, CPB1, CELA3B, SYCN, GATM, SLC4A4, DPEP1 | <https://www.ncbi.nlm.nih.gov/pmc/articles/PMC4278897/> |
| SCGN | <https://www.ncbi.nlm.nih.gov/pmc/articles/PMC5510001/> |
| AMY2A, CDKN1C | <https://www.ncbi.nlm.nih.gov/pmc/articles/PMC3582140/> |
| CFTR, GP2, CPA1, CEL, PNLIP | <https://www.ncbi.nlm.nih.gov/pmc/articles/PMC5494890/> |
| TFF1 | <https://www.ncbi.nlm.nih.gov/pmc/articles/PMC4319540/> |
| SPRINK1 | <https://www.ncbi.nlm.nih.gov/pmc/articles/PMC3097947/> |
| AQP8 | <https://www.ncbi.nlm.nih.gov/pubmed/11254497> |
| CLDN10 | <https://www.ncbi.nlm.nih.gov/pmc/articles/PMC3288608/> |
| CHGA | <https://viacyte.com/wp-content/uploads/Kelly_Nature_Biotech_07_2011.pdf> |
| PECAM1 | <http://jcs.biologists.org/content/118/18/4103> |
| MMP2 | <https://www.ncbi.nlm.nih.gov/pubmed/15734845> |
| C3 | <https://www.cell.com/cell-metabolism/pdfExtended/S1550-4131(18)30574-6> |
| REG1A | <https://bmcendocrdisord.biomedcentral.com/articles/10.1186/1472-6823-12-13> |

# **Table S6: Group I and Group II benchmark list.**

| **Dataset** | **Performance** | | **Gene Selection** |
| --- | --- | --- | --- |
| Dendritic | Group I | HEG | |
| MGE | Group I | HEG | |
| HSCs | Group I | HEG | |
| Intestinal | Group I | HEG | |
| MEM-T | Group I | HEG | |
| Pancreatic | Group I | HEG | |
| PBMC | Group I | HEG | |
| DC | Group I | HEG | |
| Myeloid | Group I | HEG | |
| H7-ESC | Group II | HEG | |
| mESCs | Group II | HEG | |
| LPS | Group II | HEG | |
| LSK | Group II | HEG | |
| HSPC | Group II | HEG | |
| Dendritic | Group I | HVG | |
| MGE | Group I | HVG | |
| HSCs | Group I | HVG | |
| Intestinal | Group I | HVG | |
| MEM-T | Group I | HVG | |
| Pancreatic | Group I | HVG | |
| PBMC | Group I | HVG | |
| DC | Group I | HVG | |
| Myeloid | Group I | HVG | |
| H7-ESC | Group I | HVG | |
| mESCs | Group I | HVG | |
| LPS | Group I | HVG | |
| LSK | Group II | HVG | |
| HSPC | Group I | HVG | |

# **Table S7:** Summary of representative scRNA-seq datasets.

| **ID** | **Year** | **Protocol** | **Source** |
| --- | --- | --- | --- |
| GSE101601 | 2017 | UMI | <https://www.ncbi.nlm.nih.gov/geo/query/acc.cgi?acc=GSE101601> |
| GSE106707 | 2017 | UMI | <https://www.ncbi.nlm.nih.gov/geo/query/acc.cgi?acc=GSE106707> |
| GSE110558 | 2018 | UMI | <https://www.ncbi.nlm.nih.gov/geo/query/acc.cgi?acc=GSE110558> |
| GSE110692 | 2018 | UMI | <https://www.ncbi.nlm.nih.gov/geo/query/acc.cgi?acc=GSE110692> |
| GSE119097 | 2018 | UMI | <https://www.ncbi.nlm.nih.gov/geo/query/acc.cgi?acc=GSE119097> |
| GSE56638 | 2014 | full length | <https://www.ncbi.nlm.nih.gov/geo/query/acc.cgi?acc=GSE56638> |
| GSE72056 | 2015 | full length | <https://www.ncbi.nlm.nih.gov/geo/query/acc.cgi?acc=GSE72056> |
| GSE81682 | 2016 | full length | <https://www.ncbi.nlm.nih.gov/geo/query/acc.cgi?acc=GSE81682> |
| GSE85527 | 2016 | UMI | <https://www.ncbi.nlm.nih.gov/geo/query/acc.cgi?acc=GSE85527> |
| GSE86977 | 2016 | full length | <https://www.ncbi.nlm.nih.gov/geo/query/acc.cgi?acc=GSE86977> |
| GSE95432 | 2017 | full length | <https://www.ncbi.nlm.nih.gov/geo/query/acc.cgi?acc=GSE95432> |
| GSE98816 | 2017 | full length | <https://www.ncbi.nlm.nih.gov/geo/query/acc.cgi?acc=GSE98816> |
| GSE95315 | 2017 | UMI | <https://www.ncbi.nlm.nih.gov/geo/query/acc.cgi?acc=GSE95315> |
| GSE95752 | 2017 | full length | <https://www.ncbi.nlm.nih.gov/geo/query/acc.cgi?acc=GSE95752> |
| GSE76381 | 2016 | UMI | <https://www.ncbi.nlm.nih.gov/geo/query/acc.cgi?acc=GSE76381> |
| GSE110679 | 2018 | UMI | <https://www.ncbi.nlm.nih.gov/geo/query/acc.cgi?acc=GSE110679> |
| GSE99888 | 2018 | UMI | <https://www.ncbi.nlm.nih.gov/geo/query/acc.cgi?acc=GSE99888> |
| GSE52529 | 2014 | full length | <http://imlspenticton.uzh.ch/robinson_lab/conquer/data-mae/GSE52529-GPL16791.rds> |
| GSE60749 | 2014 | full length | <http://imlspenticton.uzh.ch/robinson_lab/conquer/data-mae/GSE60749-GPL13112.rds> |
| GSE63818 | 2015 | full length | <http://imlspenticton.uzh.ch/robinson_lab/conquer/data-mae/GSE63818-GPL16791.rds> |
| GSE71982 | 2015 | full length | <http://imlspenticton.uzh.ch/robinson_lab/conquer/data-mae/GSE71982.rds> |
| GSE57872 | 2014 | full length | <http://imlspenticton.uzh.ch/robinson_lab/conquer/data-mae/GSE57872.rds> |
| GSE102299 | 2017 | UMI | <https://www.ncbi.nlm.nih.gov/geo/query/acc.cgi?acc=GSE102299> |
| GSE48968 | 2014 | full length | <http://imlspenticton.uzh.ch/robinson_lab/conquer/data-mae/GSE48968-GPL13112.rds> |
| GSE104157 | 2017 | full length | <https://www.ncbi.nlm.nih.gov/geo/query/acc.cgi?acc=GSE104157> |
| GSE100426 | 2017 | full length | <https://www.ncbi.nlm.nih.gov/geo/query/acc.cgi?acc=GSE100426> |
| GSE62270 | 2015 | UMI | <http://imlspenticton.uzh.ch/robinson_lab/conquer/data-mae/GSE62270-GPL17021.rds> |
| GSE106540 | 2018 | UMI | <https://www.ncbi.nlm.nih.gov/geo/query/acc.cgi?acc=GSE106540> |
| GSE81076 | 2016 | UMI | <http://imlspenticton.uzh.ch/robinson_lab/conquer/data-mae/GSE81076-GPL18573.rds> |
| SRP073808 | 2016 | full length | <http://imlspenticton.uzh.ch/robinson_lab/conquer/data-mae/SRP073808.rds> |
| EMTAB2805 | 2015 | full length | <http://imlspenticton.uzh.ch/robinson_lab/conquer/data-mae/EMTAB2805.rds> |
| GSE94383 | 2017 | full length | <http://imlspenticton.uzh.ch/robinson_lab/conquer/data-mae/GSE94383.rds> |

# **Table S8:** Summary of benchmarks used in trajectory inference

**obtained from** <https://zenodo.org/record/1443566#.XP86LtNKiRc>

| **Dataset** | **Alias** |
| --- | --- |
| aging-hsc-old_kowalczyk.rds | HSC-old |
| aging-hsc-young_kowalczyk.rds | HSC-young |
| cell-cycle_buettner.rds | mESC |
| developing-dendritic-cells_schlitzer.rds | Develop-dc |
| germline-human-female-weeks_li.rds | F-embryo |
| germline-human-male-weeks_li.rds | M-embryo |
| hematopoiesis-gates_olsson.rds | HSC-gate |
| human-embryos_petropoulos.rds | H-embryo |
| myoblast-differentiation_trapnell.rds | Myoblast |
| NKT-differentiation_engel.rds | Pan-alpha |
| pancreatic-alpha-cell-maturation_zhang.rds | NKT |
| psc-astrocyte-maturation-glia_sloan.rds | Glia |
| psc-astrocyte-maturation-neuron_sloan.rds | Neuron |
| stimulated-dendritic-cells-LPS_shalek.rds | LPS-DC |
| stimulated-dendritic-cells-PAM_shalek.rds | PAM-DC |
| stimulated-dendritic-cells-PIC_shalek.rds | PIC-DC |
| pancreatic-beta-cell-maturation_zhang.rds | Pan-beta |
| mesoderm-development_loh.rds | mesoderm |
