# Supplementary material for: scBFA: modeling detection patterns to mitigate technical noise in large-scale single-cell genomics data
Source: Genome Biol. 2019 Sep 9;20:193. doi: 10.1186/s13059-019-1806-0 (PMC6734238; doi:10.1186/s13059-019-1806-0)
Supplement: Supplementary file 2 — Review history. (DOCX 3950 kb) [file 13059_2019_1806_MOESM2_ESM.docx]

Review History

**First round of review**

**Reviewer 1**

**Are you able to assess all statistics in the manuscript, including the appropriateness of statistical tests used?**

Yes: The choice of statistical methodology is sound and suitable for the purpose.

**Comments to author:**

In this article, Li and Quon argue that for scRNA-seq data sets with high levels of technical noise, as well as scATAC-seq data, dimension reduction based only on feature detection patterns (i.e., whether any reads from a feature are observed in a given cell) gives better results than dimension reduction methods based on the estimated abundance levels. They also provide a method, BFA, to perform dimension reduction based on the feature detection values.

The method is implemented in R and available as a package from https://github.com/quon-titative-biology/scBFA. I was able to install the package [however, the installation instructions in the vignette are currently wrong since the package is submitted, but not yet accepted in Bioconductor, so maybe it would be useful to add instructions to install the package from GitHub to the README in the meanwhile] and run the example provided in the vignette without problems. The implementation nicely uses well-established Bioconductor classes for data representation and the package appears easy to run also on real data.

Overall, I found the manuscript well written and pleasant to read, and the Methods section is clear and comprehensive. Below I provide a few comments that I think would strengthen the manuscript.

1. The authors discuss the situations where BFA may perform better (or worse) than the alternative approaches and provide guidelines for when to use it, using both simulated and real data. These evaluations are generally informative, and I appreciate that the authors also illustrate situations where their method may not be suitable. What was not clear to me was how the two evaluations are connected. For example, how 'realistic' are the simulated data matrices, and where do different types of real data sets (e.g. from different platforms) fall on the spectrum of simulated data? Similarly, where would the simulated data sets fall in Figure 1c-d? I think that establishing this link would make the guidelines for application to real data sets stronger, and I think it is important to show that the simulated data indeed show similar characteristics as experimental data sets.

2. Recently, two preprints [1-2] have suggested alternatives to 'traditional' dimension reduction of scRNA-seq using log(CPM+1)+PCA, claiming improved suitability for sparse count data. While I understand that these preprints were likely not available at the time of submission of this manuscript, it would be very useful for the community if the BFA approach could be contrasted to these recent proposals.

3. For consistency between methods, feature selection was done by selecting the top 2,000 variable genes (using the count information). With a high gene detection rate, the matrix of gene detection patterns has almost no zeros, and thus contains little information. Could it be more informative, in these cases, to use another feature selection approach for BFA (based on detection patterns instead of/in addition to abundance estimates)? In particular, since the variance calculations are done on the count values (unless I'm misreading the sentence "We calculate the gene-specific variance in the gene count space"), highly expressed genes will tend to be selected, which will reduce the number of zeros in the matrix. Since the performance of BFA depends strongly on the gene detection rate, I'm also wondering what the effect would be of just selecting fewer genes for the most sparse data sets, and perhaps more genes for the dense ones, so as to aim at getting data sets with a 'fixed' gene detection rate. Would this benefit BFA, and/or other methods? I think this should be discussed, since the selection of 2,000 genes is an arbitrary choice, and the 'optimal' number may depend on the platform as well as the species.

4. The 'detection rates' shown in Figure 1a are a bit confusing, since they go up to 100%. While the reason for this (only the top 2,000 variable genes are considered as the 'full' data set) is described in the Methods, I think it would be worth mentioning this also in the figure legend to avoid misunderstandings.

5. In Figure 1d, why is the dispersion shown as a function of the TPM rather than the count (given that the dispersion is a property of the count distribution)?

6. There are two 'c' panels in Figure 1.

[1] Hafemeister & Satija: Normalization and variance stabilization of single-cell RNA-seq data using regularized negative binomial regression. bioRxiv doi: 10.1101/576827 (2019)

[2] Townes, Hicks, Aryee & Irizarry: Feature selection and dimension reduction for single cell RNA-Seq based on a Multinomial model. bioRxiv doi: 10.1101/574574 (2019).

**Reviewer 2**

**Are you able to assess all statistics in the manuscript, including the appropriateness of statistical tests used?**

Yes: The statistics is appropriate

**Comments to author:**

In this manuscript, Li and Quon report that in single-cell RNA-seq analysis dimension reduction using only the gene detection measurements is superior to methods that use both gene detection and quantification measurements. They found that their gene detection-based method BFA is fast and leads to better cell type identification and more accurate recovery of cell type-specific markers. Simulations were used to show that gene detection-based method is superior to quantification-based methods when quantification noise exceeds detection noise. They also demonstrated the applicability of BFA to single-cell ATAC-seq data.

The finding that using gene detection alone leads to better dimension reduction and cell type identification is interesting for single-cell genomic analysis. However, this conclusion is based on comparing BFA with only a few methods which do not necessarily represent the state-of-the-art. In order to convincingly show the superiority of BFA, additional benchmark analyses should to be provided to compare with the state-of-the-art methods. Also, BFA has three tuning parameters. How the method's performance depends on different choices of tuning parameters should be systematically evaluated to facilitate robust application of BFA.

Major:

1. In the scRNA-seq analysis, BFA is only compared with PCA, ZINB-WaVE and scImpute. Other studies have shown that several recent methods such as scVI and SAVER can provide more accurate quantification of gene expression, but these methods are not compared here. It is possible that scVI or SAVER imputed values can lead to better dimension reduction and cell type identification. Without comparing with these state-of-the-art methods, one cannot convincingly show the superiority of BFA.

2. In the scATAC-seq analysis, BFA is only compared with Binary PCA, PCA and Scasat. The state-of-the-art methods such as chromVAR, SCRAT and scABC are not compared. The authors mentioned that some of these methods are not compared because they require motif signals and therefore the comparison would be unfair. This argument is not very convincing. If the ultimate goal is to have the best dimension reduction and cell type identification, one should consider all methods that can achieve this goal rather than limiting oneself to methods that only use binary raw data. Many methods use motif signals or known pathway information because they can convert the sparse and binary scATAC-seq data into continuous signals which improves the power for distinguishing between different cell types. Without comparing with these methods, the usefulness of BFA in scATAC-seq analysis is unclear.

3. In BFA, eps_1, eps_2 and eps_3 are described as three tunable parameters. However, how to tune these parameters is not described. Currently, they are all set to preset values (eps_0/G, eps_0/N and eps_0/G where eps_0 = max{N, G}. How does the performance of BFA change as one tunes eps_1, eps_2 and eps_3?

Minor:

4. Two plots in Figure 1 are labeled as (c), and no plot has label (d).

5. Typo in section "Balance of technical noise explains relative performance …": in the first paragraph, "skin-in" should be "spike-in".

**Reviewer 3**

**Are you able to assess all statistics in the manuscript, including the appropriateness of statistical tests used?**

Yes: Statistics used seemed appropriate

**Comments to author:**

This paper introduces the binary factor analysis (BFA) method for dimensionality reduction of scRNA-seq and scATAC-seq data. The authors explore the data characteristics where BFA outperforms other methods and show that this approach is faster and more scalable to larger data sets. They provide the R package scBFA that allows other researchers to apply this method, which ensures it can be widely used.

Comments and questions:

I wondered whether binary detection patterns are useful for other downstream analysis tasks or only for dimensionality reduction? Perhaps this could be discussed. Also whether first applying state-of-the-art normalization or imputation methods to the data matrix followed by BFA would lead to even better performance?

For a dataset with one large, relatively homogenous group of cells, how would BFA perform? Gene dispersion would presumably be lower than for datasets with many diverse cell types. I guess I'm wondering how it would perform on a null case.

Are gene-wise dispersion estimates calculated taking group information provided by the cell labels into account, or do you assume a null model (i.e. one large group per dataset?) This wasn't clear to me from the methods.

'Supplementary Figures 3-6 illustrate the significant improvement of 2D visualizations achieved by BFA in the Group I datasets.' I really struggled to see this - adding some further commentary to the Figure labels might benefit readers like me who struggle with such plots.

Motivation for choosing PCA, ZINB-WaVE, scImpute to compare with BFA was unclear. Presumably there are many other approaches that could be compared to. Can this choice be better justified in the text?

Minor points:

The name of the new approach used in this manuscript (BFA) would be worth including in the abstract or introduction.

Results: typo in 'Balance of technical noise ...' section. 'skin-in' -> 'spike-in'

Methods: what does MAP dispersion estimates versus final estimates refer to?

There is mention that the performance gains can be incorporated into existing analyses with one line of R code via binary PCA, which sounds very convenient. Can this line of code be mentioned in the Methods (is there a function in scBFA that does this?)

For the full BFA method, the link to the code given in the paper should be corrected to https://github.com/quon-titative-biology/scBFA if this is the right URL. The implementation of this approach in an R package should also be noted in either the Results or Methods, along with some more detail about the functions a potential user would need to know about. Is there a check as part of the analysis to assess whether the quantification noise exceeds the detection noise so that a user will know whether applying BFA is likely to provide better results?

Fix reference formatting in Methods for 'LPS dataset (lane2017)'

Typo in 'Identification of marker genes' section - missing has: 'whether a gene been detected'

Potential formatting issue in 'Timing experiments' section? 'Due to the nonconvex nature of ZINBWAVE's' -> ZINB-WaVE's or are you referring to software name here which does't have the same formatting?

Typo in ATAC-seq results section 'are used predict cell' (missing 'to')

Reference 17 doesn't have any authors - please check formatting

Figure 1 c label appears twice - I assume the second should appear as d.

Check the year for dataset GSE106540 in Supp Table 2 (2244?)

**Authors Response**

**Point-by-point responses to the reviewers’ comments:**

*We thank the reviewers for their helpful comments and concerns. Please find below our summary of new experiments, as well as a point-by-point response to reviewers.*

*Summary of new experiments*

*1. Figures 1, 3, 4, Supplementary Figures 9-10: We have re-performed all scRNA-seq benchmarking experiments using a different gene selection procedure (HVG) to show that our results (scBFA superior performance on real datasets, scBFA superior performance when detection noise is less than count noise) hold under a completely different gene selection procedure that selects different sets of genes (Supplementary Table 2). As a result, we also made an interesting discovery that our original gene selection procedure (HEG) that is not widely used in the literature yields consistently better cell type identification performance compared to the HVG selection procedure that is canonical.*

*2. Figure 6, Supplementary Figure 20: Using a very recently published (April 2019) platform, dynverse, for benchmarking trajectory inference methods, we now show scBFA improves the state-of-the-art trajectory inference methods, compared to all other dimensionality reduction methods we test in our manuscript (ZINB-WaVE, PCA, scImpute, SAVER, scrna2019, sctransform, scVI). More specifically, we adapted the best-in-class method reported by the dynverse paper, Slingshot, to use each of the tested dimensionality reduction methods instead of PCA (default), and showed replacing PCA by scBFA led to the overall best performance on three performance metrics across 18 benchmark datasets.*

*3. Figure 7, Supplementary Figure 21-22: We have expanded the scATAC-seq comparison to include state-of-the-art methods Destin and scABC.*

*4. Figures 1, 3-6, Supplementary Figures 5, 9-13, 15, 19, 20: We have included additional methods for cell type identification (scrna2019, sctransform, SAVER) as well as an additional state of the art deep learning based method (scVI), and shown scBFA outperforms all of them across our experiments.*

*5. Supplementary Figure 4: We varied the hyperparameters epsilon of scBFA over four orders of magnitude to show robustness of scBFA performance to hyperparameter selection.*

*6. Supplementary Figures 6-8: We performed a series of experiments to demonstrate that our simulation framework generates realistic datasets. For each of three benchmarks (LPS, HSPC, LSK), we trained the simulation framework on the real dataset, simulated a new dataset of the same size as the original benchmark, then showed that when visualized together, cells cluster by cell type (and not by dataset of origin, e.g., real versus simulated conditions).*

*7. Figure 1, Supplementary Figures 9-10: We now show scBFA performance (under HVG selection) is robust to the choice of the simulation hyperparameter r.*

*8. Supplementary Figure 14: We now show that imputation via either SAVER or scImpute before input to scBFA makes performance of scBFA worse.*

*9. Supplementary Figure 15: We now run scBFA and other methods on the “null” case where the dataset only consists of one cell type, in order to test each method’s ability to minimize technical variation.*

*10. Supplementary Figure 23, 24: We now show Binary PCA performance is superior under both HEG and HVG selection.*

--------------

--------------

Reviewer #1

--------------

--------------

The method is implemented in R and available as a package from https://github.com/quon-titative-biology/scBFA. I was able to install the package [however, the installation instructions in the vignette are currently wrong since the package is submitted, but not yet accepted in Bioconductor, so maybe it would be useful to add instructions to install the package from GitHub to the README in the meanwhile] and run the example provided in the vignette without problems. The implementation nicely uses well-established Bioconductor classes for data representation and the package appears easy to run also on real data.

*We have updated the README to address this.*

--------------

The authors discuss the situations where BFA may perform better (or worse) than the alternative approaches and provide guidelines for when to use it, using both simulated and real data. These evaluations are generally informative, and I appreciate that the authors also illustrate situations where their method may not be suitable. What was not clear to me was how the two evaluations are connected. For example, how 'realistic' are the simulated data matrices, and where do different types of real data sets (e.g. from different platforms) fall on the spectrum of simulated data? Similarly, where would the simulated data sets fall in Figure 1c-d? I think that establishing this link would make the guidelines for application to real data sets stronger, and I think it is important to show that the simulated data indeed show similar characteristics as experimental data sets.

*We have now performed additional experiments in which we demonstrate that the simulation framework generates scRNA-seq data similar in global characteristics to actual experimental datasets (Supplementary Figures 6-8). More specifically, for the LPS, HSPC and LSK benchmarks, we independently fit our simulation framework to that benchmark, then simulated the exact same number of cells as was in the original benchmark. Upon performing dimensionality reduction and visualization of both the benchmark and corresponding simulated cells together, we found that cells of the same type clustered together, regardless of whether they were from the benchmark or from the simulation. This suggests our simulation framework generates realistic datasets.*

*With respect to making a direct link between the simulation results (scBFA should be used when detection noise is lower than count noise) and real datasets, we are currently unable to do so. This is chiefly because it is currently not possible to directly estimate and distinguish detection and count noise in real data (which is what motivated us to perform the simulations). As we show in our results, the available methods for even indirectly estimating detection noise, such as ZINB-WaVE (through its inference of which zeros are technical versus biological), do not work well. Even for experiments in which e.g. ERCC controls were added to help estimate technical noise, in high throughput datasets (e.g. those collected on microfluidic platforms like 10X) it is typically added too late in the protocol to allow estimation of all types of technical noise.*

*Despite these limitations, we still offer practical guidelines for when downstream users should apply scBFA versus count-based methods. More specifically, based on our results on the 14 real benchmark datasets (Figure 3a,b,d), we show that GDR and gene-wise dispersion are readily-estimated quantities that strongly discriminate datasets in which scBFA perform well or poorly, relative to count-based methods. Our scBFA R package provides a function, diagnose(), that puts a user’s dataset onto Figure 3a,b in order to predict whether scBFA should be applied or not.*

--------------

Recently, two preprints [1-2] have suggested alternatives to 'traditional' dimension reduction of scRNA-seq using log(CPM+1)+PCA, claiming improved suitability for sparse count data. While I understand that these preprints were likely not available at the time of submission of this manuscript, it would be very useful for the community if the BFA approach could be contrasted to these recent proposals.

*We have expanded our comparisons to scrna2019 and sctransform (the two packages that implement the methods these preprints point to), as well as an additional state of the art deep learning based method (scVI), and show that scBFA outperforms all of these methods (Figures 1, 3-6, Supplementary Figures 5, 9-13, 15, 19, 20).*

--------------

For consistency between methods, feature selection was done by selecting the top 2,000 variable genes (using the count information). With a high gene detection rate, the matrix of gene detection patterns has almost no zeros, and thus contains little information. Could it be more informative, in these cases, to use another feature selection approach for BFA (based on detection patterns instead of/in addition to abundance estimates)? In particular, since the variance calculations are done on the count values (unless I'm misreading the sentence "We calculate the gene-specific variance in the gene count space"), highly expressed genes will tend to be selected, which will reduce the number of zeros in the matrix. Since the performance of BFA depends strongly on the gene detection rate, I'm also wondering what the effect would be of just selecting fewer genes for the most sparse data sets, and perhaps more genes for the dense ones, so as to aim at getting data sets with a 'fixed' gene detection rate. Would this benefit BFA, and/or other methods? I think this should be discussed, since the selection of 2,000 genes is an arbitrary choice, and the 'optimal' number may depend on the platform as well as the species.

*We agree with the reviewer that our original gene selection procedure (we now termed HEG in the paper) will bias towards highly expressed genes, which in turn can yield little information. We liked the idea of varying the number of genes to achieve a target detection rate as the reviewer suggests, but opted instead for repeating our experiments and selecting highly variable genes (HVGs) instead. HVG selection identifies the top genes that are overdispersed (exhibit more variance than expected). HVG gene selection is quite common (as it is implemented as part of the default processing pipeline in highly cited packages such as Seurat), so we thought it important to try that gene selection procedure. We also agree with the reviewer that 2,000 genes is arbitrary, but we chose 2,000 because it is the default for e.g. Seurat, and there doesn’t seem to be community-wide agreement on a strategy to pick the number of genes a priori.*

*(Figures 1, 3, 4, Supplementary Figures 9-10) illustrate our performance using HVG selection, and (Figures 3, Supplementary Figures 5, 11-13) illustrate our performance using HEG selection. We found that HVG selection improves the relative performance of scBFA, and have replaced Figures 1, 3 and 4 (from the original manuscript) with new ones illustrating performance using HVG instead of HEG selection. We replaced the original figures because HVG seems to be a gold standard for gene selection in the community (e.g. Seurat), and HEG seems to not be used nearly as frequently.*

*We showed in Figure 3 that with respect to cell type identification, HEG selection actually improves the performance of every method we tested across almost every dataset (except for scBFA for 3/14 datasets). We now include a discussion of why we observe this trend (we think HVG selection biases towards genes that are poorly expressed but overdispersed, which probably in turn biases against cell type marker genes, which should be highly expressed in the cells they represent).*

--------------

The 'detection rates' shown in Figure 1a are a bit confusing, since they go up to 100%. While the reason for this (only the top 2,000 variable genes are considered as the 'full' data set) is described in the Methods, I think it would be worth mentioning this also in the figure legend to avoid misunderstandings.

*We have revised the legend of Figure 3 to reflect that the detection rates are calculated after performing gene selection. The revised legend for Figure 3 is pasted below:*

*Figure 3: Relative scBFA performance is positively correlated with dataset size and high technical noise. (a) Gene detection rate as a function of the number of cells for the fourteen benchmarks, calculated after either HVG or HEG selection. Group I benchmarks refers to those datasets in which scBFA is a top performer, and Group II benchmarks refers to datasets in which scBFA is a poor performer. Note that each of the fourteen benchmarks are represented twice (once under each of HEG and HVG selection). Supplementary Table 5 indicates the membership of each benchmark within Group I and Group II.*

--------------

In Figure 1d, why is the dispersion shown as a function of the TPM rather than the count (given that the dispersion is a property of the count distribution)?

*We agree that dispersion is a function of count distribution. In the specific case of Figure 1d, because we are comparing mean-dispersion trends across multiple diverse datasets, we fit dispersion to TPM to make all datasets more comparable and avoid patterns driven by differences in library size or other nuisance factors.*

--------------

There are two 'c' panels in Figure 1.

*This has been corrected.*

--------------

--------------

Reviewer #2

--------------

--------------

The finding that using gene detection alone leads to better dimension reduction and cell type identification is interesting for single-cell genomic analysis. However, this conclusion is based on comparing BFA with only a few methods which do not necessarily represent the state-of-the-art. In order to convincingly show the superiority of BFA, additional benchmark analyses should to be provided to compare with the state-of-the-art methods. Also, BFA has three tuning parameters. How the method's performance depends on different choices of tuning parameters should be systematically evaluated to facilitate robust application of BFA.

*We have included additional methods for cell type identification (scrna2019, sctransform, SAVER) as well as an additional state of the art deep learning based method (scVI), and shown scBFA outperforms all of them across our experiments (Figures 1, 3-6, Supplementary Figures 5, 9-13, 15, 19, 20).*

*We have also varied the hyperparameters epsilon of scBFA over four orders of magnitude to show robustness of scBFA performance to hyperparameter selection (Supplementary Figure 4).*

--------------

Major:

1. In the scRNA-seq analysis, BFA is only compared with PCA, ZINB-WaVE and scImpute. Other studies have shown that several recent methods such as scVI and SAVER can provide more accurate quantification of gene expression, but these methods are not compared here. It is possible that scVI or SAVER imputed values can lead to better dimension reduction and cell type identification. Without comparing with these state-of-the-art methods, one cannot convincingly show the superiority of BFA.

*We have included additional methods for cell type identification (scrna2019, sctransform, SAVER) as well as an additional state of the art deep learning based method (scVI), and shown scBFA outperforms all of them across our experiments (Figures 1, 3-6, Supplementary Figures 5, 9-13, 15, 19, 20).*

--------------

2. In the scATAC-seq analysis, BFA is only compared with Binary PCA, PCA and Scasat. The state-of-the-art methods such as chromVAR, SCRAT and scABC are not compared. The authors mentioned that some of these methods are not compared because they require motif signals and therefore the comparison would be unfair. This argument is not very convincing. If the ultimate goal is to have the best dimension reduction and cell type identification, one should consider all methods that can achieve this goal rather than limiting oneself to methods that only use binary raw data. Many methods use motif signals or known pathway information because they can convert the sparse and binary scATAC-seq data into continuous signals which improves the power for distinguishing between different cell types. Without comparing with these methods, the usefulness of BFA in scATAC-seq analysis is unclear.

*We have expanded the scATAC-seq comparison to include state-of-the-art methods Destin and scABC (Figure 7, Supplementary Figure 21-22).*

*We were unable to run SCRAT because the R package produces a Shiny web page that cannot accept as input a large number of BAM files. When running SCRAT, we obtain the error message ”Disconnect from the server” after inputting as few as 17 BAM files, whereas our datasets have up to almost 2000 BAM files. Others (such as the authors of Destin) have also noted this limitation had prevented them from benchmarking against SCRAT, see: https://academic.oup.com/bioinformatics/advance-article/doi/10.1093/bioinformatics/btz141/5367832 .*

*We were also unable to run chromVAR on our benchmark dataset, because the computeDeviations() function in the chromVAR R package often outputs NA entries in place of deviation scores, therefore preventing downstream analysis. This issue is known to the chromVAR authors, who suggest applying more aggressive filtering for either cells or peaks to reduce the number of NAs (see https://github.com/GreenleafLab/chromVAR/issues/27). When we followed these instructions, we ended up with too few cells (less than half of the cells compared to other methods) to analyze.*

*Instead of running chromVAR, we ran Destin (published on March 1, 2019). In their paper, the authors of Destin benchmarked Destin against ChromVar, scABC and Scasat, and yielded superior performance. Therefore, we think Destin reflects the state-of-art performance of analysis tools developed for scATAC-seq and is a reasonable alternative benchmark to chromVAR.*

--------------

3. In BFA, eps_1, eps_2 and eps_3 are described as three tunable parameters. However, how to tune these parameters is not described. Currently, they are all set to preset values (eps_0/G, eps_0/N and eps_0/G where eps_0 = max{N, G}. How does the performance of BFA change as one tunes eps_1, eps_2 and eps_3?

*We have now varied the hyperparameters epsilon of scBFA over four orders of magnitude to show robustness of scBFA performance to hyperparameter selection (Supplementary Figure 4).*

Minor:

--------------

4. Two plots in Figure 1 are labeled as (c), and no plot has label (d).

*This has been corrected.*

--------------

5. Typo in section "Balance of technical noise explains relative performance …": in the first paragraph, "skin-in" should be "spike-in".

*This has been corrected.*

--------------

--------------

Reviewer #3

--------------

--------------

I wondered whether binary detection patterns are useful for other downstream analysis tasks or only for dimensionality reduction? Perhaps this could be discussed.

*Using a very recently published (April 2019) platform, dynverse, for benchmarking trajectory inference methods, we now show scBFA improves the state-of-the-art trajectory inference methods, compared to all other dimensionality reduction methods we test in our manuscript (ZINB-WaVE, PCA, scImpute, SAVER, scrna2019, sctransform, scVI) (Figure 6, Supplementary Figure 20). More specifically, we adapted the best-in-class method reported by the dynverse paper, Slingshot, to use each of the tested dimensionality reduction methods instead of PCA (default), and showed replacing PCA by scBFA led to the overall best performance in three performance metrics across 18 benchmark datasets.*

--------------

Also whether first applying state-of-the-art normalization or imputation methods to the data matrix followed by BFA would lead to even better performance.

*We now show that imputation via either SAVER or scImpute before input to scBFA makes performance of scBFA worse across our benchmarks (Supplementary Figure 14).*

--------------

For a dataset with one large, relatively homogenous group of cells, how would BFA perform? Gene dispersion would presumably be lower than for datasets with many diverse cell types. I guess I'm wondering how it would perform on a null case.

*We have now included in the results a comparison of all methods in the case of performing dimensionality reduction on an ERCC-based dataset that represents a null case as described by the reviewer (one large homogeneous group of cells). In this dataset, ERCC synthetic spike-in RNAs were diluted to a single concentration (1:10) and loaded into the 10x platform in place of biological cells during the generation of the GEMs. This dataset therefore only consists of a single “cell type”, with only technical variation present (since the spike-in RNAs were diluted to the same concentration). Supplementary Figure 15 illustrates that scBFA yields a low-dimensional embedding with minimal variation between “cells” compared to the other methods. This is consistent with the intuition that methods that are less impacted by technical variation should yield less variation in the embedding space since all variation is driven by technical factors in this ERCC dataset.*

--------------

Are gene-wise dispersion estimates calculated taking group information provided by the cell labels into account, or do you assume a null model (i.e. one large group per dataset?) This wasn't clear to me from the methods.

*Gene-wise dispersion estimates are calculated across the entire dataset (each dataset is one group). We have clarified this in the methods section.*

-------------

'Supplementary Figures 3-6 illustrate the significant improvement of 2D visualizations achieved by BFA in the Group I datasets.' I really struggled to see this - adding some further commentary to the Figure labels might benefit readers like me who struggle with such plots.

*The plots are difficult to discern because we had chosen datasets for which there are many cell types (and therefore many colors). We have now replaced Supplementary Figures 2-3 with plots in which the differences are easier to discern. Also, Figure 1 and Supplementary Figure 5 should be the primary metric by which we evaluate the improvement in low dimensional embeddings with respect to cell type identification, the visualization is less impartial in that respect.*

--------------

Motivation for choosing PCA, ZINB-WaVE, scImpute to compare with BFA was unclear. Presumably there are many other approaches that could be compared to. Can this choice be better justified in the text?

*Note we have now expanded the set of tested methods to seven (PCA, ZINB-WaVE, scVI, scrna2019, sctransform, SAVER, scImpute). These methods were selected to represent diverse classes of approaches to data analysis, including dimensionality reduction methods (PCA, ZINB-WaVE, scVI), recent alternative preprocessing approaches that can be applied before dimensionality reduction (sctransform, scrna2019), and imputation methods that can be applied before dimensionality reduction (SAVER, scImpute). Within the dimensionality reduction methods, PCA is chosen because of its continued popularity (it is implemented in popular packages such as Seurat), and scVI is a leading deep learning-based dimensionality reduction method. ZINB-WaVE is chosen specifically because it is a recently-developed method and scBFA is designed as a gene detection-based analog of ZINB-WaVE, and so comparison of scBFA versus ZINB-WaVE is the most fair comparison of gene detection versus quantification based approaches.*

*This has now been clarified in the Methods section.*

--------------

Minor points:

The name of the new approach used in this manuscript (BFA) would be worth including in the abstract or introduction.

*This has been corrected.*

--------------

Results: typo in 'Balance of technical noise ...' section. 'skin-in' -> 'spike-in'

*This has been corrected.*

--------------

Methods: what does MAP dispersion estimates versus final estimates refer to?

*We have clarified in the text, how we use DEseq2 dispersion estimates (and what they mean). Here we paste the revised methods section below:*

*We use the DEseq225 package to obtain gene-specific dispersion estimates for each dataset, where dispersion is measured across all cells in a dataset. Within the DEseq2 pipeline, gene-wise dispersions are first estimated, a trend line is fit to the gene-wise dispersion estimates, and finally shrinkage is applied to the gene-wise dispersion estimates (MAP estimates). In Figure 3b, we extracted the fitted gene-wise dispersion estimates from the trend line (second step), and we fit these dispersion estimates by local linear regression (LOESS) using the gene-wise mean of transcripts per million (TPM) across all cells as the explanatory variable. To address the border effect of LOESS, we removed the top and bottom 2.5% of genes as ranked by TPM. Note that using the MAP dispersion estimates (final DEseq2 step) or the fitted dispersion estimates from the trended fit (second step) does not materially change our conclusion. The exception is for the dataset PBMC where there are 455 genes with its MAP dispersion estimates staying at their initialized value of 1e-8 during optimization, while their fitted dispersion estimates are substantially different. We therefore chose the fitted dispersion estimates to generate Figure 3b.*

--------------

There is mention that the performance gains can be incorporated into existing analyses with one line of R code via binary PCA, which sounds very convenient. Can this line of code be mentioned in the Methods (is there a function in scBFA that does this?)

*We have added a section to the Methods to describe the R command (BinaryPCA()) to run Binary PCA to perform these commands for users (either on the original data matrix, or on Seurat objects ) in R, and have also written out the full R statement in the Methods section. The corresponding methods section are pasted below:*

*Binary PCA model and calculation of the gene detection pattern matrix*

*Binary PCA describes our fast approximation to scBFA by simply running PCA, with the exception of converting the input count matrix into a detection matrix by converting non-zero values to one. We implemented Binary PCA through the addition of a single line of R code. Suppose that countMatrix is the name of the matrix in R that stores e.g. the UMI counts for each gene in each cell. To run Binary PCA, we first convert the countMatrix into the gene detection pattern matrix before running PCA via the R command:*

*countMatrix[ which(countMatrix>0) ] <- 1;*

*We then call PCA using the following command in R:*

*PCA_results <- prcomp(countMatrix, center=TRUE, scale=FALSE);*

*Note that typically scale is set to TRUE when calling PCA. For Binary PCA, we set it to FALSE because variance in gene detection is potentially associated with cell types (e.g. genes with higher detection variance are more likely to be marker genes, and therefore should contribute more to the embedding).*

--------------

For the full BFA method, the link to the code given in the paper should be corrected to https://github.com/quon-titative-biology/scBFA if this is the right URL. The implementation of this approach in an R package should also be noted in either the Results or Methods, along with some more detail about the functions a potential user would need to know about.

*We have updated the URL to point more specifically to https://github.com/quon-titative-biology/scBFA. We also now mention in the Methods section that the R package is available on GitHub, and have pointed users to the main function to call scBFA. Because of the lengthy documentation of the R package, we have provided a vignette in the R package to help users apply the package to their own data.*

--------------

Is there a check as part of the analysis to assess whether the quantification noise exceeds the detection noise so that a user will know whether applying BFA is likely to provide better results?

*With respect to making a direct link between the simulation results (scBFA should be used when detection noise is lower than count noise) and real datasets, we are currently unable to do so. This is chiefly because it is currently not possible to directly estimate and distinguish detection and count noise in real data (which is what motivated us to perform the simulations). As we show in our results, the available methods for even indirectly estimating detection noise, such as ZINB-WaVE (through its inference of which zeros are technical versus biological), do not work well. Even for experiments in which e.g. ERCC controls were added to help estimate technical noise, in high throughput datasets (e.g. those collected on microfluidic platforms like 10X) it is typically added too late in the protocol to allow estimation of all types of technical noise.*

*Despite these limitations, we still offer practical guidelines for when downstream users should apply scBFA versus count-based methods. More specifically, based on our results on the 14 real benchmark datasets (Figure 3a,b,d), we show that GDR and gene-wise dispersion are readily-estimated quantities that strongly discriminate datasets in which scBFA perform well or poorly, relative to count-based methods. Our scBFA R package provides a function, diagnose(), that puts a user’s dataset onto Figure 3a,b in order to predict whether scBFA should be applied or not.*

--------------

Fix reference formatting in Methods for 'LPS dataset (lane2017)'

*This has been corrected.*

--------------

Typo in 'Identification of marker genes' section - missing has: 'whether a gene been detected'.

*This has been corrected.*

--------------

Potential formatting issue in 'Timing experiments' section? 'Due to the nonconvex nature of ZINBWAVE's' -> ZINB-WaVE's or are you referring to software name here which doesn't have the same formatting?

*This has been corrected.*

--------------

Typo in ATAC-seq results section 'are used predict cell' (missing 'to')

*This has been corrected.*

--------------

Reference 17 doesn't have any authors - please check formatting

*This has been corrected.*

--------------

Figure 1 c label appears twice - I assume the second should appear as d.

*This has been corrected.*

--------------

Check the year for dataset GSE106540 in Supp Table 2 (2244?)

*This has been corrected.*

**Second round of review**

**Reviewer 1**

The authors have done a thorough job addressing my original concerns, and I have no further major comments. There are a couple of details:

- Figure 3a: it was very hard to discriminate between the two red colors (to me, it looks like there are five dark and only one light red point)

- "Execution of scRNA-seq analysis methods" section, ine 57: seting -> setting

- There are two Methods sections named "Execution of scRNA-seq analysis methods"; the latter refers to the scATAC-seq analysis.

- "Computing mean and dispersion curves" section: DEseq2 -> DESeq2

**Reviewer 2**

I appreciate the authors efforts on addressing some of my previous questions. However, I do not think that all my questions have been addressed, and the revised manuscript also has some new issues. Before these issues can be addressed, I do not feel that the manuscript is of sufficient quality for publication.

Major issues:

1. Many statements in the manuscript are confusing or self-conflicting. For example, in Results Section

(2):

(i) "GDR is the average fraction of genes that are detected as expressed in a given cell, and includes technical dropout events (genes whose expression is not detected, although they are truly expressed) as well as genes truly not expressed in a cell."

However, based on the authors definition of gene detection (the identification of the set of all genes truly expressed in a given cell), dropouts and genes truly not expressed should not be counted as expressed genes.

(ii) "For scBFA specifically, HVG only outperformed HEG for the three datasets with highest GDR (Fig. 3c), consistent with our intuition that scBFA performs best when GDR is lower and therefore technical noise is higher."

Why HVG outperforming HEG in high GDR datasets implies that scBFA performs best when GDR is lower?

(iii) "Across all benchmarks, we found that scBFA outperforms count-based methods for benchmarks with low GDR and high gene-wise dispersion (Fig. 3)."

No where in Figure 3 can I find a direct comparison between scBFA and count-based methods. Figure 3 mainly compared HEG and HVG. Did you mean Figure 1 instead of Figure 3? Also, one should state whether the conclusion here is based on HVG or HEG selection.

2. If "HEG selection consistently leads to higher performance in cell type identification for all methods" as stated by the authors, then why should one still use HVG? It seems that in practice one should use HEG (at least for cell type identification), and therefore it does not make sense to show the HVG results in the main manuscript but put the HEG results in the supplementary figures. It makes more sense to move the HEG results from Supplementary Figure 5 to the main paper and move the HVG results in the current Figure 1 to the supplementary.

3. The scATAC-seq comparison lacking SCRAT and chromVAR is not convincing. The other methods such as Scasat, Destin and scABC use binary count at each locus as features which are noisy, and therefore their results may not be optimal.

The authors' reason for not comparing SCRAT is that its online version cannot handle big input data. However, SCRAT has a standalone version provided in its paper (https://github.com/zji90/SCRAT) which can be installed as an R package and run in users' own computers (see SCRAT Installation instructions on the github page), just as how scBFA is installed as an R package from github. The standalone version can handle much larger datasets. Thus, the reason cited here and in the Destin paper for not comparing SCRAT is incorrect.

The authors' reason for not comparing chromVAR is that they have to apply more aggressive filtering for either cells or peaks to reduce the number of NAs, and this will result in less than half of the cells compared to other methods. However, for many application, having fewer cells but cleaner data is not a problem. One can still identify major cell clusters and infer correct trajectory. Thus, a convincing comparison with the state-of-the-art should include chromVAR. For example, one can run a separate analysis in which one applies more stringent filters to all methods to get similar number of cells to chromVAR, and then compare different methods.

Since both SCRAT and chromVAR use motif or pathway to combine data from multiple loci, the features produced by them should be cleaner for clustering and other analysis compared to the binary features used by Scasat, Destin and scABC. Therefore, both these methods need to be compared in order to see where scBFA really stands relative to the existing state-of-the-art.

**Authors Response**

**Point-by-point responses to the reviewers’ comments:**

*We thank the reviewers for their helpful comments and concerns. Please find below our summary of new experiments, as well as a point-by-point response to reviewers.*

*-----------------------------------*

*Summary of new experiments*

*-----------------------------------*

*- Figure 7, Supplementary Figure 21-24: We have expanded the scATAC-seq comparison to include the state-of-the-art methods chromVar and SCRAT.*

-----------------------------------

Reviewer #1

-----------------------------------

1. Figure 3a: it was very hard to discriminate between the two red colors (to me, it looks like there are five dark and only one light red point)

*> This has been corrected.*

2. "Execution of scRNA-seq analysis methods" section, ine 57: seting -> setting

*> This has been corrected.*

3. There are two Methods sections named "Execution of scRNA-seq analysis methods"; the latter refers to the scATAC-seq analysis.

*> This has been corrected.*

4. "Computing mean and dispersion curves" section: DEseq2 -> DESeq2

*> This has been corrected.*

-----------------------------------

Reviewer #2

-----------------------------------

1. (i) "GDR is the average fraction of genes that are detected as expressed in a given cell, and includes technical dropout events (genes whose expression is not detected, although they are truly expressed) as well as genes truly not expressed in a cell." However, based on the authors definition of gene detection (the identification of the set of all genes truly expressed in a given cell), dropouts and genes truly not expressed should not be counted as expressed genes.

*> We corrected this line to read “GDR is the average fraction of genes that are detected as expressed in a cell”.*

1. (ii) "For scBFA specifically, HVG only outperformed HEG for the three datasets with highest GDR (Fig. 3c), consistent with our intuition that scBFA performs best when GDR is lower and therefore technical noise is higher." Why HVG outperforming HEG in high GDR datasets implies that scBFA performs best when GDR is lower?

*> Figure 3c indicates that across most datasets, HEG performs at least as well as HVG, and oftentimes performs better, regardless of the underlying method used. However, there are clearly three datasets (H7-ESC, mESCs, HSPC) for which scBFA specifically performs much better under HVG selection compared to HEG selection. Figure 3d indicates those three datasets are the ones for which HEG selection leads to the highest GDR across all datasets and gene selection criterions tested, and certainly is higher than the GDR for the same datasets but under HVG selection. The relatively high GDR of H7-ESC, mESCs, HSPC under HEG versus HVG selection therefore is one possible explanation of the poor performance of HEG in those three datasets. To clarify this, we have revised the text to read “For scBFA specifically, the three benchmarks for which HVG outperformed HEG correspond to the benchmarks for which HEG selection led to the highest GDR, and HVG selection led to low GDR (Fig. 3c,d). The poor performance of scBFA combined with HEG selection can therefore be explained by the high GDR.”.*

1. (iii) "Across all benchmarks, we found that scBFA outperforms count-based methods for benchmarks with low GDR and high gene-wise dispersion (Fig. 3)." No where in Figure 3 can I find a direct comparison between scBFA and count-based methods. Figure 3 mainly compared HEG and HVG. Did you mean Figure 1 instead of Figure 3? Also, one should state whether the conclusion here is based on HVG or HEG selection.

*> Based on Figure 1 and Supplementary Figure 5, we identified datasets in which scBFA is a top performer in terms of cell type classification (“Group I” datasets), as well as datasets in which scBFA is not the top performer (“Group II” datasets), across both HEG and HVG selection procedures. Using these labels to indicate datasets in which scBFA is a top performer, Figure 3 illustrates that Group I datasets tend to have low GDR and high gene-wise dispersion. Note Figure 3 puts both HEG and HVG into the same plot, so we are treating each individual benchmark under HVG and HEG selection as two distinct data points in the scatterplots with different characteristics (GDR and genewise dispersion). We have now clarified this in the text, which now reads “Across all benchmarks and gene selection criteria, we found that scBFA outperforms count-based methods for benchmarks with low GDR and high gene-wise dispersion (Figs. 1 and 3, Group I benchmarks)”.*

2. If "HEG selection consistently leads to higher performance in cell type identification for all methods" as stated by the authors, then why should one still use HVG? It seems that in practice one should use HEG (at least for cell type identification), and therefore it does not make sense to show the HVG results in the main manuscript but put the HEG results in the supplementary figures. It makes more sense to move the HEG results from Supplementary Figure 5 to the main paper and move the HVG results in the current Figure 1 to the supplementary.

*> We agree with the reviewer that it is somewhat controversial whether to put HEG or HVG into the main figure. We ultimately decided to put the HVG results in the main figures because HVG is the canonical gene selection procedure used almost universally across all scRNA-seq analysis papers we have read, certainly far more than those papers using HEG selection. We therefore concluded that from the readers’ perspective, showing the performance gain that can be realized using scBFA in their current pipelines (that use HVG selection) is more impactful than showing results based on HEG selection. We do have text in the results and discussion arguing that HEG should be used more frequently in the context of cell type identification.*

3. The scATAC-seq comparison lacking SCRAT and chromVAR is not convincing. The other methods such as Scasat, Destin and scABC use binary count at each locus as features which are noisy, and therefore their results may not be optimal. The authors' reason for not comparing SCRAT is that its online version cannot handle big input data. However, SCRAT has a standalone version provided in its paper (https://github.com/zji90/SCRAT) which can be installed as an R package and run in users' own computers (see SCRAT Installation instructions on the github page), just as how scBFA is installed as an R package from github. The standalone version can handle much larger datasets. Thus, the reason cited here and in the Destin paper for not comparing SCRAT is incorrect. The authors' reason for not comparing chromVAR is that they have to apply more aggressive filtering for either cells or peaks to reduce the number of NAs, and this will result in less than half of the cells compared to other methods. However, for many application, having fewer cells but cleaner data is not a problem. One can still identify major cell clusters and infer correct trajectory. Thus, a convincing comparison with the state-of-the-art should include chromVAR. For example, one can run a separate analysis in which one applies more stringent filters to all methods to get similar number of cells to chromVAR, and then compare different methods. Since both SCRAT and chromVAR use motif or pathway to combine data from multiple loci, the features produced by them should be cleaner for clustering and other analysis compared to the binary features used by Scasat, Destin and scABC. Therefore, both these methods need to be compared in order to see where scBFA really stands relative to the existing state-of-the-art.

*> We have now modified our application of chromVAR to throw out the generated NAs as well as run SCRAT’s R package, and shown scBFA outperforms both chromVAR and SCRAT across all scATAC-seq benchmarks (Figure 6, Supplementary Figure 21-24).*

**Third round of review**

**Reviewer 2:**

The authors have addressed all my questions.
